# New and Redesigned pRS Plasmid Shuttle Vectors for Genetic Manipulation of *Saccharomyces cerevisiae*

**DOI:** 10.1534/g3.111.001917

**Published:** 2012-05-01

**Authors:** Mark K. Chee, Steven B. Haase

**Affiliations:** Department of Biology, Duke University, Durham, North Carolina 27708

**Keywords:** *Saccharomyces cerevisiae*, plasmid shuttle vector, auxotrophic marker, drug resistance marker, polylinker/multiple cloning site

## Abstract

We have constructed a set of 42 plasmid shuttle vectors based on the widely used pRS series for use in the budding yeast *Saccharomyces cerevisiae* and the bacterium *Escherichia coli*. This set of pRSII plasmids includes new shuttle vectors that can be used with histidine and adenine auxotrophic laboratory yeast strains carrying mutations in the genes *HIS2* and *ADE1*, respectively. Our pRSII plasmids also include updated versions of commonly used pRS plasmids from which common restriction sites that occur within their yeast-selectable biosynthetic marker genes have been removed to increase the availability of unique restriction sites within their polylinker regions. Hence, our pRSII plasmids are a complete set of integrating, centromere and 2μ episomal plasmids with the biosynthetic marker genes *ADE2*, *HIS3*, *TRP1*, *LEU2*, *URA3*, *HIS2*, and *ADE1* and a standardized selection of at least 16 unique restriction sites in their polylinkers. Additionally, we have expanded the range of drug selection options that can be used for PCR-mediated homologous replacement using pRS plasmid templates by replacing the G418-resistance *kan*MX4 cassette of pRS400 with MX4 cassettes encoding resistance to phleomycin, hygromycin B, nourseothricin, and bialaphos. Finally, in the process of generating the new plasmids, we have determined several errors in existing publicly available sequences for several commonly used yeast plasmids. Using our updated sequences, we constructed pRS plasmid backbones with a unique restriction site for inserting new markers to facilitate future expansion of the pRS series.

The budding yeast *Saccharomyces cerevisiae* is an important and widely used model system for studying eukaryotic cell biology that has also become important in the new fields of functional genomics and systems biology (Botstein and Fink 2011). Among the most important tools available for the genetic manipulation of *S. cerevisiae* are plasmid shuttle vectors, which can be used in both *S. cerevisiae* and the bacterium *Escherichia coli* (Da Silva and Srikrishnan 2011; Iserentant 1990). In addition to an antibiotic resistance marker and a bacterial replication origin for propagation in *E. coli*, these shuttle vectors contain a second yeast-selectable marker. The latter marker is typically either a gene that confers resistance to antibiotics or antifungal toxins (Van Den Berg and Steensma 1997) or a biosynthetic gene that rescues an auxotrophic mutation (Pronk 2002). Today, most commonly encountered *S. cerevisiae* shuttle vectors belong to one of three classes (Da Silva and Srikrishnan 2011; Iserentant 1990; Romanos *et al.* 1992): (1) integrating plasmids (YIp), which lack yeast replication origins, must be inserted into the yeast genome in order to be replicated; (2) centromere plasmids (YCp), which contain both a yeast centromere (*CEN*) and an autonomously replicating sequence (*ARS*), are replicated in yeast at single or very low copy number; or (3) yeast episomal plasmids (YEp), which contain a 2μ circle replication origin and the *cis*-acting *STB* (stability) locus (Mehta *et al.* 2002), are replicated autonomously in yeast at high copy number.

Among the shuttle vectors most frequently used today by researchers working with *S. cerevisiae* are the YXplac series (Gietz and Sugino 1988) and the pRS series (Brachmann *et al.* 1998; Christianson *et al.* 1992; Sikorski and Hieter 1989). The systematic design and utility of these plasmids have inspired the construction of similar plasmid sets for use in other fungal model organisms (Adams *et al.* 2005; Chen 1996; Gould *et al.* 1992). The YXplac series is based on the plasmid pUC19 (Gietz and Sugino 1988), whereas the pRS series uses a hybrid backbone built using either the pBluescript or pBluescriptII polylinker/multiple cloning site (MCS) ligated to the pBluescribe replication origin (Christianson *et al.* 1992; Sikorski and Hieter 1989). Compared with older and larger pBR322-based yeast vectors (Botstein *et al.* 1979; Kuo and Campbell 1983; Tschumper and Carbon 1980), both the YXplac and pRS series offer important advantages of small plasmid sizes (<7 kb), high copy number in bacteria, a good range of unique sites for cloning, the capacity for blue-white screening, and a range of yeast-selectable markers. These markers include the *S. cerevisiae* biosynthetic genes *TRP1*, *LEU2*, and *URA3*, which can be used with almost all commonly encountered laboratory strains that are auxotrophic for tryptophan, leucine, or uracil, respectively. However, there are limitations to both series with respect to histidine and adenine auxotrophy. First, the YXplac series does not include prototrophic markers that can be used in strains that are either His^−^ and/or Ade^−^ (Gietz and Sugino 1988). Second, although the pRS series does include plasmids marked with either *HIS3* (Christianson *et al.* 1992; Sikorski and Hieter 1989) or *ADE2* (Brachmann *et al.* 1998), not all common laboratory strains that are His^−^ and/or Ade^−^ carry *HIS3* and/or *ADE2* mutations; the strains BF264-15D (abbreviated as 15D) (Reed *et al.* 1985), J17 (Fitzgerald-Hayes *et al.* 1982), and B93 (Vezhinet *et al.* 1991) are examples of *his2ade1* mutants. Given that auxotrophic markers are important for facilitating the genetic manipulation of *S. cerevisiae* (Pronk 2002), the inability to conveniently exploit all the available auxotrophic markers in a given laboratory strain is an unfortunate limitation.

Our need to exploit the *his2* mutation in 15D inspired the construction of the integrating plasmid pRS306H2 (Chee and Haase 2010). Despite its utility, pRS306H2 suffers from an acute shortage of unique sites in its MCS. This highlights another shortcoming of the pRS series plasmids. The choice of restriction sites for cloning constructs into pRS plasmids is marker-dependent and may complicate *in vitro* cloning. This is due to the presence of several restriction sites within the *S. cerevisiae HIS3*, *TRP1*, *LEU2*, *URA3* and other yeast-selectable marker sequences of the pRS plasmids that are also found in the pBluescript/pBluescript II MCS of the pRS plasmid backbone (Brachmann *et al.* 1998; Eriksson *et al.* 2004; Sikorski and Hieter 1989). This is in contrast to the YXplac series (Gietz and Sugino 1988) in which *TRP1*, *LEU2*, and *URA3* markers were mutagenized to remove restriction sites in common with the pUC19 MCS. Hence, all 10 of the 6-bp restriction sites in the pUC19 MCS are unique in every YXplac plasmid (Gietz and Sugino 1988). If constrained by restriction site availability, an investigator seeking to integrate a construct of interest into the yeast genome using a particular pRS plasmid would have to first clone a given construct into another integrating plasmid with a different marker or into an episomal plasmid by using recombination-mediated/gap-repair methods (Ma *et al.* 1987; Oldenburg *et al.* 1997) before moving it into the integrating plasmid with the desired marker using *Pvu*I or *Bss*HII fragment exchange (Brachmann *et al.* 1998; Sikorski and Hieter 1989). However, given the additional labor and time required, this may not be an ideal solution for everyone.

In addition to introducing genetic constructs into yeast and *in vivo* cloning by homologous recombination, the pRS series of vectors can also be used for PCR-mediated homologous replacement of sequences in the budding yeast genome (Baudin *et al.* 1993; Brachmann *et al.* 1998; Replogle *et al.* 1999). This method allows for sequences in the *S. cerevisiae* genome to be replaced by a selectable marker amplified by PCR with 5′ and 3′ flanking sequences matching the sequences upstream and downstream of the sequence of interest (Baudin *et al.* 1993; Lorenz *et al.* 1995; Wach 1996). The simplicity and utility of PCR-mediated gene replacement has led to its usage in other fungal model organisms as well (Kaur *et al.* 1997; Walther and Wendland 2008; Wendland *et al.* 2000). Due to the standardized design of the pRS series, a single pair of oligonucleotide primers can be used to amplify any prototrophic marker from any pRS plasmid (Brachmann *et al.* 1998) for transforming yeast.

Heterologous dominant drug resistance markers, such as the *kan*MX module that confers resistance to G418 (Wach *et al.* 1994), provide some advantages over prototrophic biosynthetic markers for PCR-mediated gene disruption/deletion. Although using a prototrophic marker requires working with a strain that carries the corresponding auxotrophic mutation, no such requirement exists for drug resistance markers. Moreover, whereas the usage of drug resistance genes is more flexible as they lack homology to the *S. cerevisiae* genome (Goldstein and McCusker 1999), prototrophic markers derived from *S. cerevisiae* work best in strains with “designer deletion alleles” (Brachmann *et al.* 1998; Replogle *et al.* 1999), in which gene conversion or rescue of the corresponding auxotrophic mutation is prevented. Finally, whereas prototrophic markers have the potential to complicate phenotypic analysis and must be carefully controlled for (Pronk 2002), drug resistance markers reportedly have neutral effects on growth under non-selective conditions (Goldstein and McCusker 1999; Hadfield *et al.* 1990).

Although plasmids that carry other MX markers, such as *hph*MX, *nat*MX, and *pat*MX (Goldstein and McCusker 1999; Hentges *et al.* 2005; Wach *et al.* 1994), have been developed using the pFA backbone, pRS400 (Brachmann *et al.* 1998) is the only pRS plasmid in the literature that carries an MX drug resistance cassette, namely, *kan*MX4 (Wach *et al.* 1994). On the other hand, the pRS series offers an unmatched selection of prototrophic markers for PCR-mediated replacement. Hence, researchers may find themselves employing two or more pairs of oligonucleotides to replace a particular gene sequence with markers from different plasmid series.

In this report, we describe our attempts to overcome the limitations described above. First, we have constructed new *HIS2*- and *ADE1*-marked shuttle vectors by replacing the yeast-selectable marker of existing pRS plasmids. In each of these new plasmid vectors, we have preserved the uniqueness of all 18 common restriction sites found in their polylinker regions, providing valuable new tools for genetic analysis in *his2* and *ade1* laboratory yeast strains.

Second, to expand the availability of unique sites in the MCS of existing pRS plasmids, we have mutagenized the *S. cerevisiae* genes *ADE2*, *HIS3*, *TRP1*, *LEU2*, and *URA3* using a strategy similar to that used during the construction of the YXplac series (Gietz and Sugino 1988). We also swapped the 2μ origin of the pRS episomal vectors with that from the YEplac series so as to remove the *Xba*I site within. Altogether, we have generated 42 pRSII plasmid shuttle vectors with 16 restriction sites in their polylinkers that are unique throughout the entire series: pRSII30x/31x/32x with the pBluescript KS= MCS (Sikorski and Hieter 1989) and pRS40x/41x/42x with the pBluescript II SK= MCS (Brachmann *et al.* 1998; Christianson *et al.* 1992). The pRSII plasmids are easier to manipulate *in vitro* than their pRS predecessors and will facilitate molecular cloning and yeast plasmid construction.

Third, we have expanded the repertoire of drug resistance cassettes available in pRS plasmids and, hence, the number of markers that can be amplified using a single pair of oligonucleotides for PCR-mediated gene replacement. We replaced the *kan*MX4 cassette in pRS400 with four drug resistance genes, derived from other commonly used plasmids (Goldstein and McCusker 1999; Gueldener *et al.* 2002), that encode resistance to the antibiotic compounds phleomycin, hygromycin B, nourseothricin, and bialaphos.

Finally, in the course of constructing our new plasmids, we have uncovered several errors in publicly accessible nucleotide sequences for existing yeast plasmids. These errors probably went unnoticed because the restriction maps for these plasmids were based on the published sequences of the different parts used to build them. Some of these errors caused restriction sites to be missed while suggesting the presence of non-existent sites. One error in the sequence for pRS402, pRS412, and pRS422 (Brachmann *et al.* 1998) is particularly serious as it fails to document the presence of a 163-bp insertion in these plasmids that causes a drastic reduction in yield when one attempts to amplify the *ADE2* marker with standard pRS primers. Another error that required rectification was the opposite orientation of the *CEN6*/*ARSH4* cassette in pRS313 and pRS413 compared to all other pRS *CEN* plasmids. We have documented the sequence discrepancies we observed to improve the accuracy of molecular cloning. Importantly, the true sequence of the *ADE2* and *LEU2* pRS vectors facilitated the construction of pRS backbone plasmids with a unique restriction site (*Bgl*II and *Age*I, respectively) located between the two pRS primer binding sites. Novel yeast-selectable markers of the user’s choice may therefore be easily introduced to construct additional pRS vectors in the future.

## Materials and Methods

### Plasmid construction

Standard techniques were used for DNA manipulation. Restriction enzymes were purchased from New England Biolabs, except for *Pfo*I, which was purchased from Fermentas. Ligations were performed using T4 DNA ligase purchased from Invitrogen. Both PCR-mediated site-directed mutagenesis and gene amplification for cloning purposes were performed using either cloned *Pfu* Turbo DNA polymerase (Stratagene) or KOD HotStart DNA polymerase (Toyobo, Novagen/EMD Chemicals). Antarctic phosphatase (New England Biolabs) was used to treat symmetrical ends of plasmids cut with a single restriction enzyme to prevent recircularization. Plasmid propagation was carried out in Invitrogen MAX Efficiency DH5α bacteria grown in lysogeny broth (LB) (Bertani 2004) supplemented with either 50–100 μg/ml ampicillin sodium salt or 10 μg/ml kanamycin sulfate purchased from Sigma-Aldrich. Bacterial transformants were selected for on LB 2% agar plates supplemented with either 100 μg/ml ampicillin sodium salt or 60 μg/ml kanamycin sulfate.

Plasmid construction details are provided in supporting information, File S1. In general, we followed the strategy employed for mutagenesis of *TRP1*, *LEU2*, and *URA3* during construction of the YXplac plasmids (Gietz and Sugino 1988). We used silent mutations that preserve the amino acid sequence to mutagenize restriction sites found in the open reading frame of the yeast-selectable auxotrophic marker genes *ADE2*, *HIS3*, *TRP1*, *LEU2*, *URA3*, *ADE1*, and *HIS2* (Table S1). As for the few sites occurring in the untranslated regions of these genes, we used neutral changes that should not affect either transcription initiation or termination. Oligonucleotides used for site-directed mutagenesis are listed in Table S2.

### *In silico* cloning

The software ApE (M. Wayne Davis, University of Utah, http://biologylabs.utah.edu/jorgensen/wayned/ape/) and pDRAW32 (Acaclone Software, http://www.acaclone.com/) were used to analyze sequence data, design primers, and design cloning strategies. Additionally, PlasMapper 2.0 (Dong *et al.* 2004) and BVTech Plasmid 5.1 (Bio Visual Tech Inc.) were used to generate the plasmid maps shown in the figures.

### Yeast strains and media

Two auxotrophic wild-type strains of budding yeast were used to verify the ability of the pRSII plasmids described in this report to rescue auxotrophic mutations. The first is 15Daub (Kaiser *et al.* 1999), a *bar1*Δ *ura3*Δ*ns* derivative of BF264-15D (*MATa ade1his2leu2-3,112 trp1-1^a^*) (Reed *et al.* 1985), abbreviated as 15D in our lab. The second is a *bar1*Δ derivative of W303a (*MATa ade2-1 his3-11,15 leu2-3,112 trp1-1 ura3-1 can1-100*) (Elion *et al.* 1993), also known as SBY688 in our lab. The prototrophic yeast strain S288C (*MAT*α *SUC2gal2 mal mel flo1flo8-1 hap1hobio1bio6*) (Mortimer and Johnston 1986), also known as SBY1806 in our lab, was used to verify the utility of our new pRS plasmids carrying MX4 drug resistance cassettes in PCR-mediated gene replacement (File S1). Yeast cultures were grown in standard YEPD medium (1% yeast extract, 2% peptone, 0.012% adenine, 0.006% uracil, and 2% dextrose), except during selection; plate media were prepared by adding 2% agar. Growth temperatures were kept between 25° and 26°. Prototrophic transformants were selected for by plating on synthetic complete dropout plates (0.67% yeast nitrogen base, 2% dextrose, 2% agar) lacking the appropriate amino acid or nucleobase. To select for drug-resistant transformants, we suggest referring to previously published protocols for guidelines (Baudin *et al.* 1993; Gatignol *et al.* 1987; Goldstein and McCusker 1999; Wenzel *et al.* 1992). Selection conditions that we have tested ourselves and suggestions for users who experience difficulty with drug selection are described in File S1.

### PCR protocol for amplifying pRS/pRSII plasmid yeast-selectable markers

Similarly to what has previously been described (Brachmann *et al.* 1998), we used primers starting with 40–50 nucleotides of gene-specific sequence at the 5′ end and followed by either 5′-CAGATTGTACTGAGAGTGC-3′ (pRS forward primer binding site) or 5′-CCTTACGCATCTGTGCGG-3′ (pRS reverse primer binding site) to amplify the yeast-selectable marker sequences in any of the pRS or pRSII plasmids; examples of primer pairs used to target the genes *KIP1*, *CIN8*, and *ADE2* are provided in Table S3. As noted before (Goldstein and McCusker 1999), PCR amplification of the *nat*MX4 and *pat*MX4 drug resistance cassettes requires the addition of 5% DMSO. The reaction parameters we employed were: 94° for 1 min followed by 34 amplification cycles (94° for 45 sec, 55° for 45 sec, 72° for 1 min/kb of expected PCR product size), 72° for 10 min. Annealing and denaturation times can be shortened to 30 sec, and the extension temperature can be reduced to 70° or 68°. *Taq* DNA polymerase (Denville) was used for marker amplification at 0.05μl.

### Yeast transformation

Yeast were transformed using high-efficiency methods involving lithium acetate, polyethylene glycol, and denatured, single-stranded salmon sperm DNA (Gietz and Schiestl 2007; Gietz and Woods 2001). To transform the wild-type strains 15Daub and W303a using pRS/pRSII plasmids, we used either 200 ng of integrating plasmid linearized by restriction at a unique site within the yeast-selectable prototrophic marker sequence or 50 ng of *CEN*/2μ plasmid. Prototrophic transformants were selected for by spinning down yeast cells after heat shocking and resuspending them in sterile water before plating on the appropriate dropout medium. For drug selection, the yeast were resuspended in YEPD and allowed to recover before plating. The transformation of yeast with PCR-amplified MX4 drug resistance cassettes is described in detail in File S1.

## Results and Discussion

### New *HIS2*-marked yeast-bacteria shuttle vectors

**Figure 1:**
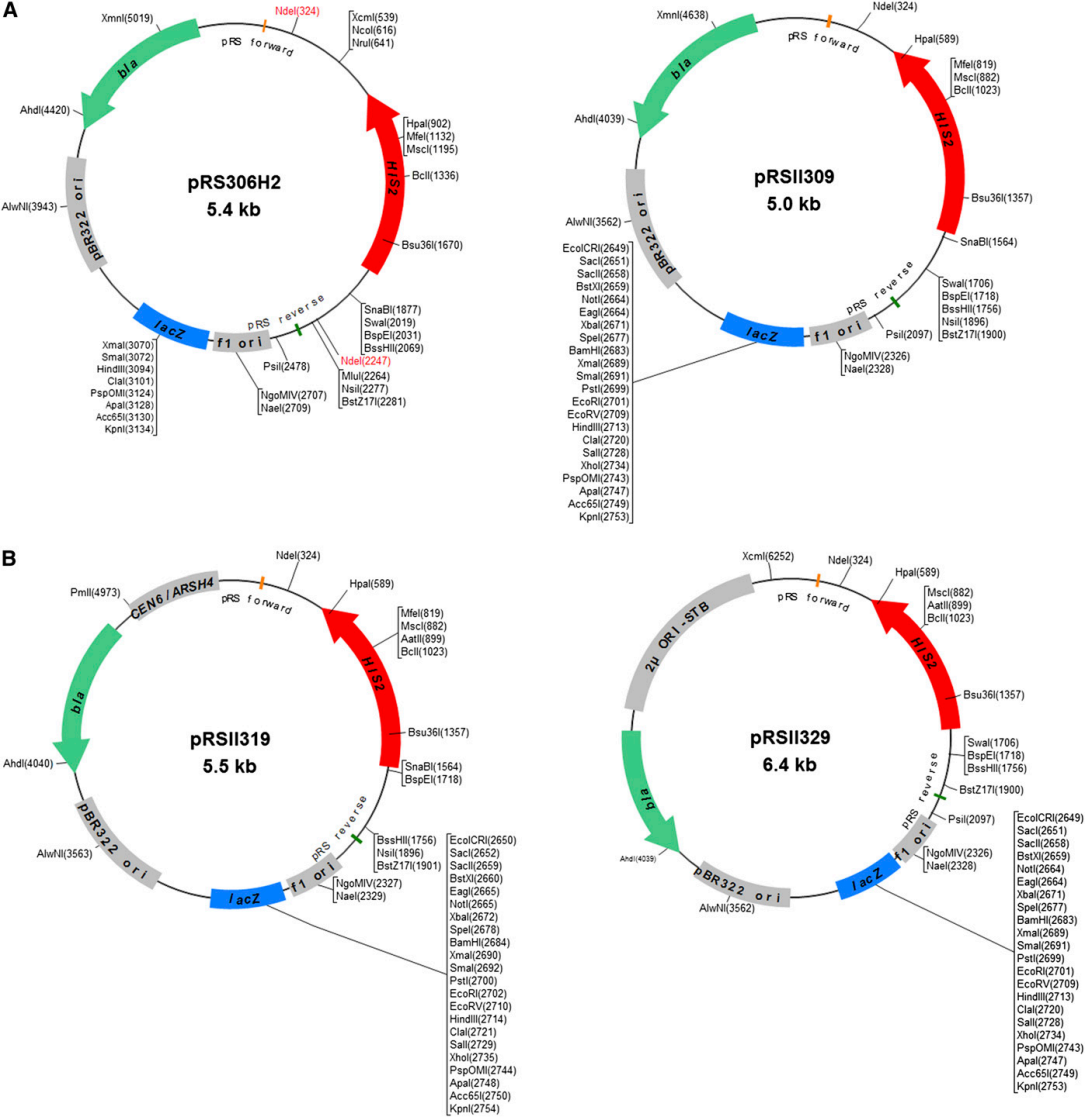
Features of new *S. cerevisiae HIS2*-marked plasmid shuttle vectors. (A) Restriction maps of the integrating plasmids pRS306H2 (Chee and Haase 2010) and pRSII309. (B) Episomal plasmids pRSII319 (*CEN*) and pRSII329 (2μ). Although the features of each plasmid are drawn to scale, the size of the maps are not scaled according to plasmid size. Aside from the two *Nde*I sites highlighted in red for pRS306H2, only unique restriction sites are shown and isoschizomers are indicated.

Whereas *S. cerevisiae HIS3* encodes imidazoleglycerol-phosphate dehydratase, *HIS2* encodes histidinolphosphatase. Both of these enzymes function in histidine biosynthesis but catalyze different steps (Alifano *et al.* 1996; Gorman and Hu 1969; Struhl and Davis 1980). Despite the availability of plasmids that can be used with histidine auxotrophic laboratory strains of budding yeast that are *his3* mutants, these plasmids cannot be used with His^−^ strains that are *his2* mutants. The latter includes include strains such as 15D (Reed *et al.* 1985), which is widely used in cell-cycle research. The comparative scarcity of *HIS2*-marked yeast vectors poses an unnecessary limitation when working with *his2* strains. Our first attempt at making an integrating vector with a *HIS2* marker involved the disruption of the *URA3* marker in pRS306 (Sikorski and Hieter 1989) with a wild-type *HIS2* allele, resulting in pRS306H2 (Chee and Haase 2010). Although this plasmid has been successfully used to both integrate genetic constructs into the *S. cerevisiae* genome (Chee and Haase 2010) and to delete genes of interest by PCR (unpublished data), it suffers from a shortage of unique sites in its MCS, contains extraneous sequences, and could be streamlined (Figure 1A). Moreover, *HIS2*-marked centromere and 2μ episomal versions of pRS306H2 have yet to be constructed.

To improve upon pRS306H2, we have completely rebuilt it using a different strategy (File S1). Using a site-directed mutagenesis strategy similar to that of Gietz and Sugino (1988), we removed the *Bam*HI and *Xho*I sites present in the wild-type *HIS2* gene (Table S1 and File S1). We subsequently used the mutagenized *HIS2*, PCR-amplified with *Nde*I and *Nsi*I ends, to replace almost the entire *URA3* gene in pRS306. The resulting plasmid, which we have dubbed pRSII309, is the updated successor to pRS306H2. pRSII309 is 0.4 kb smaller than its predecessor due mostly to the near-complete excision of the *URA3* marker from pRS306 (Figure 1); in pRS306H2, the *URA3* marker was disrupted between the *Nco*I and *Nsi*I sites. Moreover, all of the 18 common restriction sites in the pRSII309 polylinker region (the pBluescript KS= MCS) are unique. This replacement method is similar to the one we used to generate pRS306H2 in that it can be used to convert other *URA3*-marked yeast plasmids to the *HIS2* marker (Chee and Haase 2010). We subsequently constructed *CEN* and 2μ episomal derivatives of pRSII309, pRSII319 and pRSII329, respectively, as well as pRSII409/419/429, which carry the pBluescript II SK= MCS (Table 1 and File S1). The significance of the pRSII designation is explained below.

**Table 1:** pRSII series plasmids

Plasmid Names	Yeast-selectable Marker	Yeast Replication Origin	MCS	Non-unique Restriction Sites Remaining in MCS	Addgene ID
pRSII302	*ADE2*	None	pBluescript KS=	*Eco*RV, *Bst*XI	35433
pRSII402	*ADE2*	None	pBluescript II SK=	*Eco*RV, *Bst*XI	35434
pRSII303	*HIS3*	None	pBluescript KS=	*Bst*XI	35435
pRSII403	*HIS3*	None	pBluescript II SK=	*Bst*XI	35436
pRSII304	*TRP1*	None	pBluescript KS=	*Eco*RV, *Bst*XI	35437
pRSII404	*TRP1*	None	pBluescript II SK=	*Eco*RV, *Bst*XI	35438
pRSII305	*LEU2*	None	pBluescript KS=	*Eco*RV, *Bst*XI	35439
pRSII405	*LEU2*	None	pBluescript II SK=	*Eco*RV, *Bst*XI	35440
pRSII306	*URA3*	None	pBluescript KS=	*Eco*RV	35441
pRSII406	*URA3*	None	pBluescript II SK=	*Eco*RV	35442
pRSII308	*ADE1*	None	pBluescript KS=	None	35443
pRSII408	*ADE1*	None	pBluescript II SK=	None	35444
pRSII309	*HIS2*	None	pBluescript KS=	None	35445
pRSII409	*HIS2*	None	pBluescript II SK=	None	35446
pRSII312	*ADE2*	*CEN6*/*ARSH4*	pBluescript KS=	*Eco*RV, *Bst*XI	35447
pRSII412	*ADE2*	*CEN6*/*ARSH4*	pBluescript II SK=	*Eco*RV, *Bst*XI	35448
pRSII313	*HIS3*	*CEN6*/*ARSH4*	pBluescript KS=	*Bst*XI	35449
pRSII413	*HIS3*	*CEN6*/*ARSH4*	pBluescript II SK=	*Bst*XI	35450
pRSII314	*TRP1*	*CEN6*/*ARSH4*	pBluescript KS=	*Eco*RV, *Bst*XI	35451
pRSII414	*TRP1*	*CEN6*/*ARSH4*	pBluescript II SK=	*Eco*RV, *Bst*XI	35452
pRSII315	*LEU2*	*CEN6*/*ARSH4*	pBluescript KS=	*Eco*RV, *Bst*XI	35453
pRSII415	*LEU2*	*CEN6*/*ARSH4*	pBluescript II SK=	*Eco*RV, *Bst*XI	35454
pRSII316	*URA3*	*CEN6*/*ARSH4*	pBluescript KS=	*Eco*RV	35455
pRSII416	*URA3*	*CEN6*/*ARSH4*	pBluescript II SK=	*Eco*RV	35456
pRSII318	*ADE1*	*CEN6*/*ARSH4*	pBluescript KS=	None	35457
pRSII418	*ADE1*	*CEN6*/*ARSH4*	pBluescript II SK=	None	35458
pRSII319	*HIS2*	*CEN6*/*ARSH4*	pBluescript KS=	None	35459
pRSII419	*HIS2*	*CEN6*/*ARSH4*	pBluescript II SK=	None	35460
pRSII322	*ADE2*	2μ *ORI-STB*	pBluescript KS=	*Eco*RV, *Bst*XI	35461
pRSII422	*ADE2*	2μ *ORI-STB*	pBluescript II SK=	*Eco*RV, *Bst*XI	35462
pRSII323	*HIS3*	2μ *ORI-STB*	pBluescript KS=	*Bst*XI	35463
pRSII423	*HIS3*	2μ *ORI-STB*	pBluescript II SK=	*Bst*XI	35464
pRSII324	*TRP1*	2μ *ORI-STB*	pBluescript KS=	*Eco*RV, *Bst*XI	35465
pRSII424	*TRP1*	2μ *ORI-STB*	pBluescript II SK=	*Eco*RV, *Bst*XI	35466
pRSII325	*LEU2*	2μ *ORI-STB*	pBluescript KS=	*Eco*RV, *Bst*XI	35467
pRSII425	*LEU2*	2μ *ORI-STB*	pBluescript II SK=	*Eco*RV, *Bst*XI	35468
pRSII326	*URA3*	2μ *ORI-STB*	pBluescript KS=	*Eco*RV	35469
pRSII426	*URA3*	2μ *ORI-STB*	pBluescript II SK=	*Eco*RV	35470
pRSII328	*ADE1*	2μ *ORI-STB*	pBluescript KS=	None	35471
pRSII428	*ADE1*	2μ *ORI-STB*	pBluescript II SK=	None	35472
pRSII329	*HIS2*	2μ *ORI-STB*	pBluescript KS=	None	35473
pRSII429	*HIS2*	2μ *ORI-STB*	pBluescript II SK=	None	35474

### New *ADE1*-marked shuttle vectors

Adenine auxotrophy presents an analogous problem to that we have described for histidine auxotrophy in *S. cerevisiae*. *S. cerevisiae ADE1* encodes N-succinyl-5-aminoimidazole-4-carboxamide ribotide synthetase, whereas *ADE2* encodes phosphoribosylaminoimidazole carboxylase, enzymes required for distinct steps in *de novo* purine biosynthesis (Jones and Fink 1982; Myasnikov *et al.* 1991; Stotz and Linder 1990). Ade^−^ strains that carry *ade1* and/or *ade2* mutations accumulate a red pigment that distinguishes them from Ade^=^ yeast, which are white (Fisher 1969; Silver and Eaton 1969). Hence, *ade1*, *ade2* as well as *ade1ade2* mutants are valuable for visual red-white screening of transformants and other color-based assays (Ugolini and Bruschi 1996; Weng and Nickoloff 1997). However, as *ADE2*-marked plasmids are not useful when working with *ade1* mutant strains, investigators would benefit from having a set of *ADE1*-marked pRS plasmids available to complement existing *ADE2* pRS plasmids (Brachmann *et al.* 1998).

**Figure 2:**
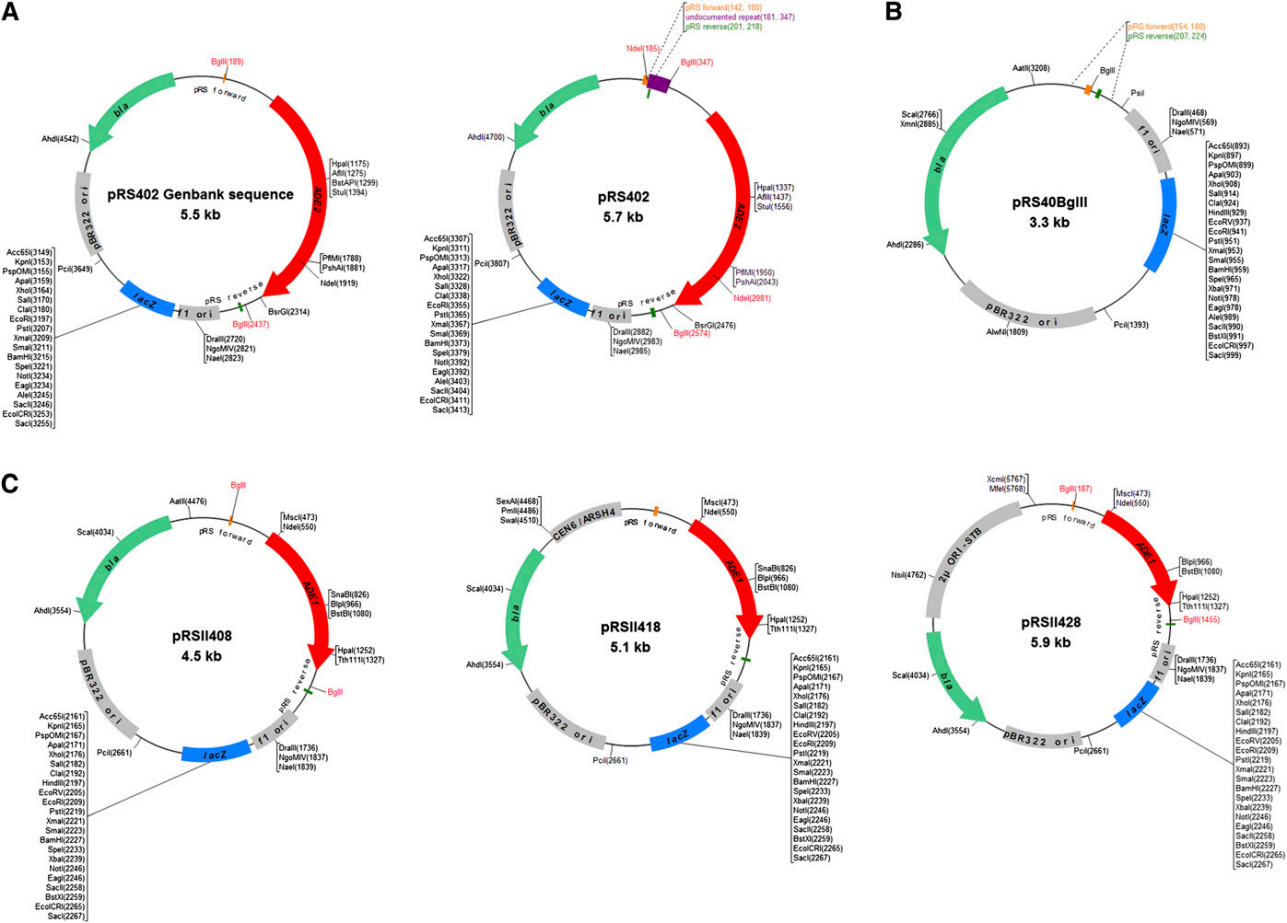
Features of existing *S. cerevisiae ADE2* and new *ADE1*-marked plasmid shuttle vectors. (A) Restriction maps of pRS402 built using existing GenBank (left) and experimentally determined (right) sequence data. A previously undocumented 163-base pair insertion indicated in dark purple; this insertion is a nearly identical repeat of 163 nucleotides 3′ of the *ADE2* marker and hence carries an extra pRS reverse primer binding site (highlighted). This repeat was removed to generate the pRS backbone plasmid pRS40BglII (B) that was subsequently used to construct pRSII402 and pRSII408. (C) Restriction maps of pRSII408, pRSII418, and pRSII428. Unique restriction sites are shown in black, and non-unique *Bgl*II and *Nde*I sites are shown in red; isoschizomers are also indicated.

Without pre-existing *ADE1* shuttle vectors in hand, we chose the *ADE2*-marked pRS402 (Brachmann *et al.* 1998) to build an *ADE1*-marked integrating plasmid. Based on its GenBank sequence (accession no. U93717.1), the *ADE2* marker in pRS402 is flanked by *Bgl*II sites and is thus easily replaced (Figure 2A); however, we discovered disagreements between the actual and the GenBank sequences of pRS402 when performing restriction analysis and Sanger sequencing. First, restricting pRS402 (Brachmann *et al.* 1998) with *Nde*I yields two fragments (1.9 and 3.8 kb) instead of the single 5.5 kb molecule predicted by its GenBank sequence. Moreover, we could not sequence pRS402 using a standard pRS reverse primer (5′-CCTTACGCATCTGTGCGG-3′) as Sanger capillary sequencing reactions consistently returned overlapping electropherograms, strongly suggesting that the primer was annealing to two different sites on the plasmid.

By sequencing with other primers (Table S4), we determined the presence of an undocumented insertion in pRS402 (Figure 2A) that contains an unwanted second pRS reverse primer binding site, which we first had to remove along with the *ADE2* marker, and then generate a pRS backbone plasmid with a unique *Bgl*II site (Figure 2A). We also mutagenized the *ADE1* gene to remove five restriction sites that are found in the pBluescript/pBluescript II MCS similarly to what we did to *HIS2* (Table S1 and File S1). Next, we subcloned the mutagenized *ADE1* marker into the *Bgl*II site to generate pRSII408 (Figure 2B). As the same unwanted insertion was found in both pRS412 and pRS422 (Brachmann *et al.* 1998), we used a similar strategy to construct pRSII418 (*CEN*) and pRSII428 (2μ) and subsequently generated pRSII308/318/328. As with their *HIS2*-marked counterparts, all 18 common restriction sites in the polylinker region of the new *ADE1* pRSII plasmids are unique (Table 1).

### A second generation of pRS plasmids (pRSII) with expanded unique restriction site selection within the polylinker region

Due to the existence of restriction sites common to both their yeast-selectable marker sequences as well as their polylinker regions, unique site selection within the MCS of current pRS plasmids is marker-dependent (Christianson *et al.* 1992; Sikorski and Hieter 1989). As shown in Table S1, only 9 of the 18 common restriction sites in the MCS of existing pRS vectors marked with either *ADE2*, *HIS3*, *TRP1*, *LEU2*, or *URA3* are unique across the board; this number drops to 7 if the *MET15*, *LYS2*, and *ADE8* markers found in other pRS series plasmids (Brachmann *et al.* 1998; Eriksson *et al.* 2004; Tomlin *et al.* 2001) are also considered (data not shown). Additionally, the 2μ pRS plasmids (Christianson *et al.* 1992) carry an *Xba*I site within the 2μ replication origin originally derived from YEp24 (Hartley and Donelson 1980). As a consequence, the *Xba*I site in the pRS42x MCS is not unique.

In contrast to the pRS series, the *S. cerevisiae TRP1*, *LEU2*, and *URA3* alleles used to construct the YXplac series of shuttle vectors were mutagenized to remove all 6-bp restriction sites that are also found in the pUC19 MCS (Gietz and Sugino 1988) of that series. Additionally, the *Xba*I site within the 2μ origin from YEp24 (Hartley and Donelson 1980) was removed before it was incorporated into the 2μ YXplac (YEplac) plasmids (Gietz and Sugino 1988). Hence, all 10 of the 6-bp sites in the pUC19 MCS (5′-*Eco*RI-*Sac*I-*Kpn*I-*Sma*I-*Bam*HI-*Xba*I-*Sal*I-*Pst*I-*Sph*I-*Hin*dIII-3′) are unique throughout the YXplac series. Removal of the *Xba*I site in the 2μ origin of YEplac195 (Gietz and Sugino 1988) as well as the non-YXplac series plasmids YEp351 and YEp352 (Hill *et al.* 1986) does not appear to significantly alter their copy number, estimated by Southern blotting, when compared to the pRS42x plasmids (Christianson *et al.* 1992; Li and Johnston 2001; Vashee and Kodadek 1995; Velmurugan *et al.* 2000).

#### Removing common restriction sites outside the MCS:

**Figure 3:**
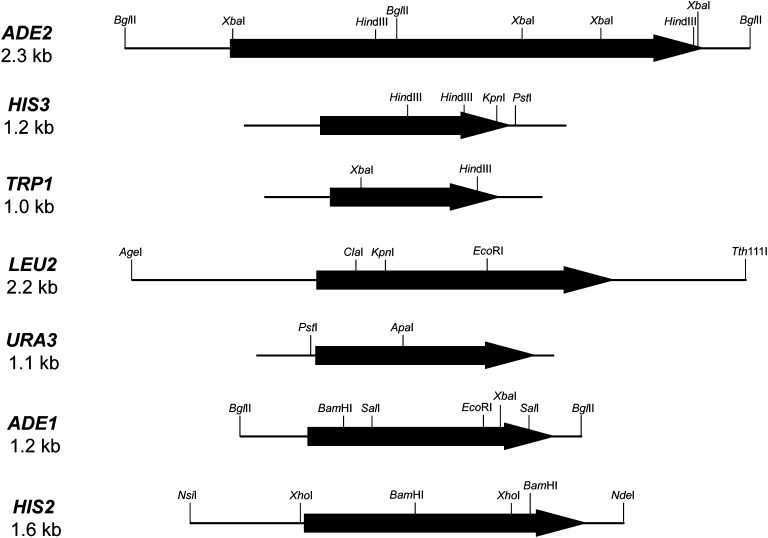
Schematic diagrams of the prototrophic biosynthetic marker genes found in pRSII series plasmids. Restriction sites in each marker that were targeted for removal before incorporation into pRSII series plasmids are indicated, as are the restriction sites that immediately flank the *ADE2*, *LEU2*, *ADE1*, and *HIS2* markers within the pRS or pRSII plasmids. The ORF in each marker is indicated by a block arrow. A complete list of pRSII plasmids is found in Table 1, and the oligonucleotides used for site-directed mutagenesis of restriction sites are found in Table S2. The *Bam*HI site found in the *ADE1* genomic sequence was previously removed from the *ADE1* allele (Nagley *et al.* 1988) used to generate pRSII408. The *Bgl*II site found in the *ADE2* genomic sequence was also previously removed (Stotz and Linder 1990) from the *ADE2* allele used to generate pRSII402. Although the *Apa*I site in *URA3* overlaps with a *dcm* methylation site, plasmid DNA isolated from DH5α *dcm^=^* bacteria is still cleaved at this site by *Apa*I. The gene diagrams shown are drawn to scale.

When building our new *HIS2* and *ADE1* shuttle vectors, we emulated the efforts of Gietz and Sugino (1988) and kept all the common restriction sites in the polylinker region unique by mutagenizing the two marker genes. We subsequently explored the feasibility of altering the prototrophic marker sequences (Figure 3) of other commonly used pRS plasmids to both increase the availability of unique sites in their polylinkers as well as to standardize unique site selection across the series. To do so in an efficient manner, we wanted to subclone the *TRP1*, *LEU2*, and *URA3* alleles developed for the YXplac series into the pRS series plasmids where convenient and separately mutagenize *HIS3* and *ADE2*. Additionally, to make the *Xba*I site within the MCS of the pRS 2μ plasmids unique, we wanted to replace their 2μ origin with that from YEplac195 (Gietz and Sugino 1988).

After the reconstruction detailed in File S1, we have reduced restriction site overlap between the five markers and the pBluescript/pBluescript II MCS to the point where 16 of the 18 common restriction sites in the polylinker region of our pRSII plasmid series are universal; *Eco*RV and *Bst*XI, one or both of which occur in all five mutagenized markers (Figure 3), were the only sites we left intact (Table 1). We did not initially plan to mutagenize the *Apa*I site in the *URA3* marker because it overlaps with a *dcm* methylation site (Larimer 1987) and most laboratory bacterial strains are *dcm^=^*; however, we found that *Apa*I is able to cleave pRS306 isolated from *dcm^=^* DH5α *E. coli* at this site (data not shown), underscoring the difficulty of predicting inhibitory effects by site-specific DNA methylation (McClelland *et al.* 1994). As a result, we removed the site altogether.

The ability of the modified prototrophic markers to rescue their corresponding auxotrophic mutations in yeast was verified by transforming the auxotrophic wild-type strains 15Daub (Kaiser *et al.* 1999) and W303a (Elion *et al.* 1993) with the new pRSII plasmids. Side-by-side transformations were done with existing pRS plasmids for comparison, except for the modified *HIS2* and *ADE1* alleles described above. For *HIS2* and *ADE1*, comparisons were made by transforming 15Daub with TA cloning plasmids containing either the unmodified or the mutagenized alleles (pGEM-T-*HIS2* and pDrive-*ADE1*, File S1), which act as yeast integrating plasmids when linearized (see *Materials and Methods*). We observed no significant differences in transformation efficiency (data not shown).

#### Correcting aberrant features in existing pRS plasmids:

In the course of building our pRSII plasmids, we discovered aberrant features in five pRS plasmids that contradict the intended uniform design of that plasmid series. We have either removed or corrected these in our pRSII series plasmids to eliminate confusion and to standardize their design. Significantly, we rebuilt the three *ADE2* pRS plasmids to remove the undocumented insertion mentioned earlier that is found in pRS402/412/422 (Figure 2A). This 163-bp insertion is a near-identical repeat of the sequence immediately flanking the 3′ end of the *ADE2* marker (162 out of the 163 nucleotides are identical) and thus contains a binding site for standard pRS reverse primers that lies between the pRS forward primer binding site and the *ADE2* marker. This extra reverse primer binding site greatly reduces the yield of any attempt to amplify the *ADE2* marker by PCR for homologous gene replacement in yeast (Figure S1).

We also determined that the *CEN6*/*ARSH4* cassette in pRS313 and pRS413 was inserted in the opposite orientation to those in other pRS *CEN* plasmids. During construction of the pRS31x plasmids (Sikorski and Hieter 1989), the authors had intended for the *CEN6*/*ARSH4* cassette to be inserted such that *CEN6* would be closest to the *bla* gene, but *CEN6* is instead closer to the *HIS3* marker in both pRS313 and pRS413. We have corrected this inconsistency during the construction of pRSII313 and pRSII413 (File S1).

#### pRSII plasmid features:

Our initial set of 42 pRSII integrating, centromere and 2μ episomal plasmids are listed in Table 1; as these plasmids will be made available through Addgene, the corresponding Addgene plasmid IDs are indicated. The naming conventions established for the pRS plasmid series (Brachmann *et al.* 1998; Christianson *et al.* 1992; Sikorski and Hieter 1989) also apply to the pRSII plasmids (Table 1 and Table S1). The elimination of common 6-bp restriction sites like *Kpn*I, *Hind*III, *Eco*RI, *and Xba*I from the seven prototrophic marker gene sequences (Figure 3) makes it more convenient to clone inserts into the pRSII polylinker and also simplifies the movement of inserts between pRSII plasmids. As has been described, a *Pvu*I digest can be used to exchange inserts between pRS300-series and between pRS400-series plasmids (Sikorski and Hieter 1989), and *Bss*HII can be used to exchange inserts only between pRS400-series plasmids (Brachmann *et al.* 1998), features that are inherent to the backbone and remain unchanged in their pRSII counterparts. As we have also maintained the characteristic uniform structure of the pRS series in our pRSII plasmids, they are compatible with the many sets of pRS-based plasmids that have been designed for uses as varied as epitope tagging, heterologous gene expression in yeast, and recombination cloning. By adapting the added features of such existing plasmids to the pRSII backbone, derivatives with a standardized MCS, differing only in the yeast-selectable marker that they carry, can easily be generated.

Users should be take note of the addendum in the original paper that described the initial set of pRS plasmids (Sikorski and Hieter 1989). The MCS of the pRS300 (and hence the pRSII300) series plasmids contains a single base pair deletion found in all of Strategene’s pBluescript KS plasmids. This deletion removed a G immediately upstream of the *Kpn*I site and downstream of the *lacZ* reporter’s ATG start codon. Blue-white screens still work (by an unknown mechanism) with the pRS300 (and pRSII300) series plasmids, but users who plan to generate LacZ fusion proteins should be aware of this frameshift. The pRS400 (and pRSII400) plasmids are not affected by this deletion as their MCS is derived from pBluescript II KS= (Sikorski and Hieter 1989).

### New pRS plasmids with drug resistance markers for PCR-mediated gene disruption/deletion

**Figure 4:**
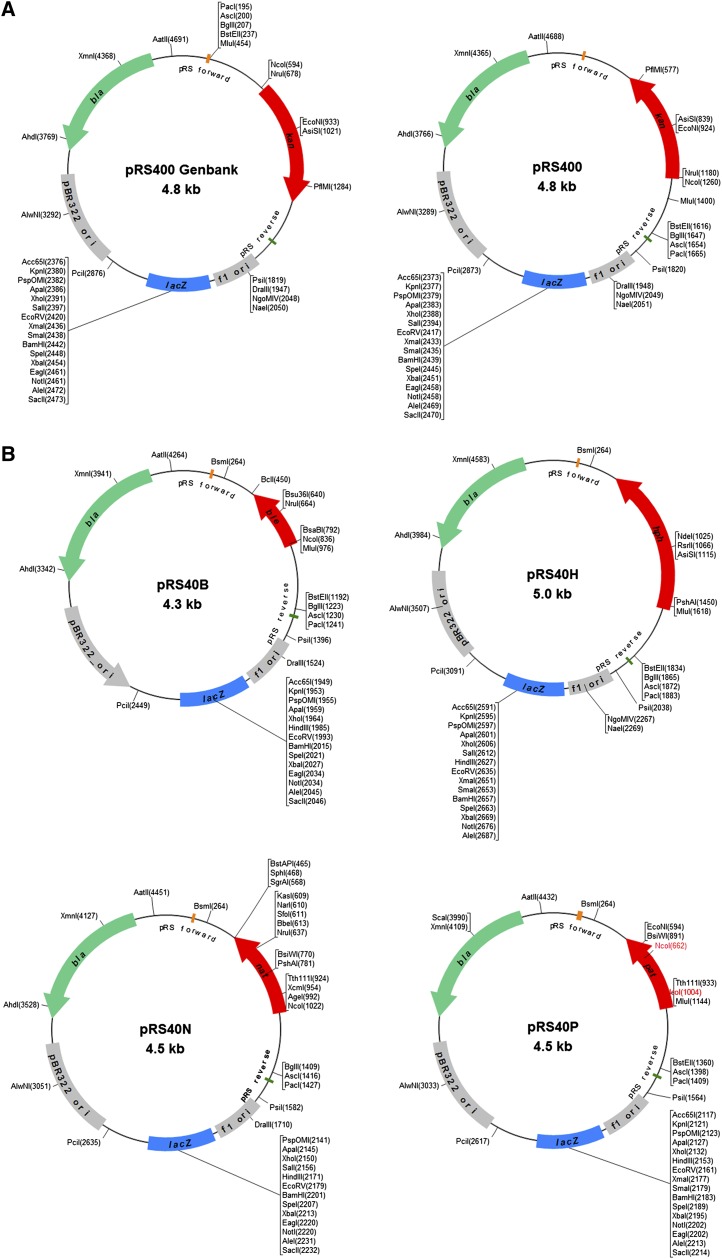
Features of pRS400 and plasmids derived from it carrying new dominant drug resistance MX4 cassettes that can be amplified by PCR for gene disruption/deletion in yeast. (A) Restriction maps of pRS400 with *kan*MX4 cassette for G418 resistance, based on existing GenBank (left) and experimentally derived (right) nucleotide sequences. The orientation of the *kan*MX4 cassette is inverted in the Genbank sequence. (B) New MX4 plasmids derived from pRS400. Top, pRS40B with *ble*MX4 cassette for phleomycin resistance (left) and pRS40H with *hph*MX4 cassette for hygromycin B resistance (right). Bottom, pRS40N with *nat*MX4 cassette for nourseothricin resistance (left) and pRS40P with *pat*MX4 cassette for bialaphos resistance (right). Unique restriction sites are shown in black, whereas the *Nco*I sites we found to be non-unique in the *pat*MX4 cassette (File S1) are shown in red; isoschizomers are also indicated.

The introduction of the plasmid pRS400 made drug selection possible for users of the pRS series seeking to either disrupt or delete sequences of interest in the budding yeast genome by PCR-mediated homologous replacement (Brachmann *et al.* 1998). pRS400 (Figure 4A) contains a heterologous *kan*MX4 module (Wach *et al.* 1994), in which the *E. coli* transposon Tn903 *kan* gene (Grindley and Joyces 1980) is under the control of the constitutive *Ashbya gossypii TEF1* promoter. Tn903 *kan* encodes aminoglycoside phosphotransferase, which confers resistance to kanamycin/G418 by phosphorylating the antibiotic (Oka *et al.* 1981). It should be noted, however, that the *kan*MX4 cassette in pRS400 is oriented in the opposite direction to what its GenBank sequence (accession no. U93713.1) indicates (Figure 4A).

To expand the repertoire of drug resistance markers in the pRS plasmid series, we replaced the *kan*MX4 cassette of pRS400 with MX4 cassettes containing drug resistance genes from other commonly used plasmids (Goldstein and McCusker 1999; Gueldener *et al.* 2002) and generated four new pRS plasmids that will also be made available through Addgene (Table 2 and Figure 4B): (1) pRS40B contains the gene *ble*, originally cloned from transposon Tn5 isolated from *Klebsiella pneumoniae*, which encodes a protein that binds with high affinity to phleomycin/bleomycin family antibiotics (Gatignol *et al.* 1987; Genilloud *et al.* 1984), such as Zeocin (Invitrogen); (2) pRS40H contains *hph* from *K. pneumoniae*, which encodes hygromycin B phosphotransferase for hygromycin B resistance (Gritz and Davies 1983); (3) pRS40N contains *nat1*, from *Streptomyces noursei*, which encodes nourseothricin N-acetyltransferase for resistance toward nourseothricin, a mixture of streptothricins (Krügel *et al.* 1993); and (4) pRS40P contains *pat*, from *Streptomyces viridochromogenes*, which encodes phosphinothricin N-acetyltransferase for resistance to bialaphos (Strauch *et al.* 1988; Wohlleben *et al.* 1988), to make pRS40P. When using the above drugs to select for yeast transformants, we recommend referring to previously suggested drug concentrations and media recipes for guidelines (Gatignol *et al.* 1987; Goldstein and McCusker 1999; Wenzel *et al.* 1992). We successfully tested our new drug resistance plasmids by targeted replacement of the *ADE2* gene (Figure S2 and Table S5) in the wild-type yeast strain S288C (Mortimer and Johnston 1986) and have included selection conditions that we used (File S1).

**Table 2:** pRS400-based drug resistance MX4 marker plasmids

Plasmid Name	Drug Resistance Gene in MX4 Cassette	Species of Origin for Resistance Gene	Source Plasmid for MX4 Cassette	Addgene ID
pRS40B	*ble*	*Klebsiella pneumoniae*, transposon Tn5	pUG66 (Gueldener *et al.*, 2002)	35478
pRS40H	*hph*	*Klebsiella pneumoniae*	pAG32 (Goldstein and McCusker 1999)	35479
pRS40N	*nat1*	*Streptomyces noursei*	pAG25 (Goldstein and McCusker 1999)	35480
pRS40P	*pat*	*Streptomyces viridochromogenes*	pAG29 (Goldstein and McCusker 1999)	35481

There are now a total of 5 drug resistance MX4 markers and 10 prototrophic markers (*MET15*, *ADE2*, *HIS3*, *TRP1*, *LEU2*, *URA3*, *LYS2*, *ADE1*, *HIS2*, and *ADE8*) that can be amplified using a single pair of oligonucleotides (examples given in Table S3) from known pRS/pRSII plasmids (Brachmann *et al.* 1998; Sikorski and Hieter 1989; Tomlin *et al.* 2001) for targeted homologous replacement in budding yeast. A suggested PCR protocol compatible with all pRS/pRSII plasmids is provided in *Materials and Methods*. As many of the drugs used for selection are compatible with minimal media (File S1), it is possible to design double selection schemes involving both nutritional and drug selection. Marker exchange within a deletion/disruption strain is also possible using the same pair of oligonucleotides; given the identical *TEF1* promoter and terminator regulatory sequences found in all MX cassettes (Wach 1996; Wach *et al.* 1994), exchanging one cassette for another is particularly efficient (Goldstein and McCusker 1999). Additionally, the absence of cross-resistance between the antibiotic resistance markers allows for strains carrying more than one marker to be selected for on media containing two or more drugs (Goldstein and McCusker 1999).

### Errors in publicly available sequences for existing yeast plasmids

In addition to the errors in existing sequences for plasmids that we described earlier in this report, we observed inconsistencies in existing sequence data available for several other yeast plasmids we worked with in the course of this study. Errors were sometimes first detected by unexpected differences observed in the number and sizes of restriction fragments; however, a large number were first determined by Sanger sequencing, such as during the construction of pRSII304 (File S1). Sequencing the pRS plasmids was necessary to verify suspected errors, as the restriction maps and sequences for the first pRS plasmids were generated based on published sequences of their components available at the time (Sikorski and Hieter 1989). Plasmids were sequenced using the oligonucleotide primers listed in Table S4, and errors were verified by sequencing related plasmids for comparison. For example, errors identified in the pRS402 GenBank sequence were verified by sequencing pRS412 and pRS422. Errors in the backbone sequence common across the pRS series were also identified in this way. Our findings reinforce sequence errors that have been reported elsewhere for pRS416 and pRS426 (Tomlin *et al.* 2001) as well as the *HIS3*-marked pRS vectors (http://genome-www.stanford.edu/vectordb/vector.html). We have included details of the most significant errors we determined in sequences deposited in public databases such as GenBank in File S1 and have listed sequences in need of updating in Table S6. Accurate sequence data will greatly benefit cloning using the affected plasmids.

### pRS backbone plasmids for generating future pRS plasmids with new yeast-selectable markers

As mentioned above, using the updated sequences for the *ADE2*-marked pRS plasmids, we were able to excise the additional undesired pRS reverse primer binding site and generate pRS backbone vectors with a unique *Bgl*II site for the insertion of new marker sequences (File S1). Similarly, the updated sequence data that we have collected has allowed us to generate a second set of pRS backbone vectors with a unique *Age*I site by excising the *LEU2* marker from *LEU2*-marked pRS plasmids using *Tth*111I and *Age*I and subsequently recircularizing the backbone (File S1); the presence of an *Age*I site flanking the *LEU2* marker in pRS305/315/405/415/425 was previously undocumented. Previous efforts to build new pRS plasmids with novel yeast-selectable markers involved lengthy cloning processes with multiple steps (Eriksson *et al.* 2004; Tomlin *et al.* 2001). With two non-overlapping sets of restriction enzymes that generate cohesive ends compatible with either *Bgl*II or *Age*I, our new backbone vectors provide greater flexibility and should simplify the future construction of pRS/pRSII plasmids with additional yeast-selectable markers. Our new backbone vectors are also intended to complement the existing markerless pRS plasmids pJK142 (integrating), pGC25 (*CEN*), and pGC26 (2μ) (Brachmann *et al.* 1998), which have a unique *Nde*I site for inserting yeast-selectable markers.

We hope that the new yeast plasmids introduced in this report as well as the updated sequences for existing plasmids will provide a sufficiently complete and cost-effective set of tools for starting research projects that employ budding yeast as a model. We also hope that they will facilitate the development of new molecular genetic tools for yeast research.

## Supplementary Material

Supporting Information
