# Supplementary material for: New and Redesigned pRS Plasmid Shuttle Vectors for Genetic Manipulation of Saccharomyces cerevisiae
Source: G3 (Bethesda). 2012 May 1;2(5):515–26. doi: 10.1534/g3.111.001917 (PMC3362935; doi:10.1534/g3.111.001917)
Supplement: Supporting Information [file supp_2_5_515__index.html]

Supporting Information 

# New and Redesigned pRS Plasmid Shuttle Vectors for Genetic Manipulation of *Saccharomyces**cerevisiae*

## Supporting Information for Chee and Haase, 2012

**Files in this Data Supplement:**

- Supporting Information - Figures S1 and S2, File S1, and Tables S1-S6 (PDF, 1.5 MB)
- File S1 - Materials & Methods (PDF, 214 KB)
- Figure S1 - PCR amplification of *ADE2* marker for targeted gene replacement with either pRSII402 or pRS402 as the template (PDF, 526 KB)
- Figure S2 - PCR amplification of MX4 markers from pRS400 and its new derivatives for targeted replacement of *ADE2* in the *S. cerevisiae* genome (PDF, 810 KB)
- Table S1 - Common restriction sites found in both yeast prototrophic marker sequences and the pBluescript/pBluescript II (PDF, 87 KB)
- Table S2 - Restriction sites targeted for removal in yeast auxotrophic marker sequences and oligonucleotide primers used for site-directed mutagenesis (PDF, 76 KB)
- Table S3 - Oligonucleotide primers used to test amplification of yeast-selectable marker sequences from pRS/pRSII plasmids (PDF, 72 KB)
- Table S4 - Oligonucleotide primers used to sequence yeast plasmids (PDF, 68 KB)
- Table S5 - Total numbers of S288C *ade2Δ* transformants obtained with MX4 casettes amplified from pRS400-derived plasmids (PDF, 85 KB)
- Table S6 - Existing publicly available sequences for yeast shuttle vectors in need of correction (PDF, 76 KB)
