# Supplementary material for: New and Redesigned pRS Plasmid Shuttle Vectors for Genetic Manipulation of Saccharomyces cerevisiae
Source: G3 (Bethesda). 2012 May 1;2(5):515–26. doi: 10.1534/g3.111.001917 (PMC3362935; doi:10.1534/g3.111.001917)
Supplement: Supporting Information [file supp_2.5.515_FileS1.pdf]

File S1

Materials & Methods

Plasmid construction

pRSII303/403 (*HIS3*) series: A 0.6 kb fragment of the *HIS3* gene containing the restriction sites targeted for mutagenesis was excised from pRS303 (Sikorski and Hieter 1989) with *Nde*I and *Nsi*I, and subsequently ligated to circularized pGEM-T (Promega) cut with the same two enzymes. By using PCR-mediated site-directed mutagenesis, we introduced silent mutations that preserve the amino acid sequence to mutagenize the *Hin*dIII and *Kpn*I sites within the *HIS3* ORF, while the *Pst*I site in the 3'UTR was mutagenized using a nucleotide transversion as it does not fall within the sequences known to be important for *HIS3* transcription termination (Mahadevan *et al.* 1997) (Table S2). The two *Hin*dIII sites were mutagenized in one pGEM-T-*HIS3* clone while the *Kpn*I and *Pst*I sites were mutagenized in a separate clone. The *HIS3* insert was sequenced in full after each mutagenesis step to verify that it was free of spurious errors. pGEM-T-*HIS3*$^{\Delta 2HindIII,\ KpnI,\ PstI}$ was generated by ligating the 1.1 kb *Nhe*I-*Alw*NI fragment from pGEM-T-HIS3$^{\Delta KpnI,\ PstI}$ to pGEM-T-*HIS3*$^{\Delta 2HindIII}$ cut with *Nhe*I and *Alw*NI.

The mutagenized *HIS3 Nde*I-*Nsi*I fragment was subcloned into pRS303 and pRS403 cut with *Nde*I and *Nsi*I to generate pRSII303 and pRSII403, respectively. pRSII313 and pRSII413 were generated by excising the 1.7 kb *Pfo*I-*Ngo*MIV fragment from pRSII303 and ligating it to pRS316 and pRS 416 (Sikorski and Hieter 1989) cut with both *Pfo*I and *Ngo*MIV. In order to construct the 2μ episomal plasmid pRSII423, first, pRS425 was digested with *Alw*NI and *Pfo*I to isolate the 2.9 kb fragment containing the 2μ replication origin of that plasmid. This fragment was ligated to pRSII403 cut with both *Alw*NI and *Pfo*I; to complete the construction of pRSII423, we replaced the 0.65kb *Sna*BI-*Mfe*I section of the 2μ origin with the *Sna*BI-*Mfe*I fragment from the 2μ origin of YEplac195 (Gietz and Sugino 1988). Although both two 2μ origin sequences were derived from the same plasmid, YEp24 (Hartley and Donelson 1980), that from YEplac195 has been modified to eliminate the *Xba*I site within (Gietz and Sugino 1988) as described in the main text. Finally, pRSII323 was made by ligating the 2.9kb *Alw*NI-*Pfo*I fragment from pRSII423 to pRSII303 digested with *Alw*NI and *Pfo*I.

pRSII304/404 (*TRP1*) series: Due to the absence of compatible restriction sites caused by the dissimilarity between the length of untranslated regions of the *TRP1* markers in the YIp/YCp/YEplac and the pRS304/404 series, the following strategy was adopted to first build pRSII304. A fragment of the *TRP1* gene spanning the mutagenized *Xba*I site was excised from YIplac204 (Gietz and Sugino 1988) with *Pml*I and *Mfe*I, and ligated to pRS304 (Sikorski and Hieter 1989) cut with the same two enzymes. The resulting plasmid, pRS304$^{\Delta XbaI}$, was subject to PCR-mediated site-directed mutagenesis to alter the *Hin*dIII site in the *TRP1* marker to the same sequence as that of the *TRP1* allele in YIplac204 (Table S1); the lack of convenient restriction sites prevented direct subcloning from YIplac204 to eliminate this *Hin*dIII site. The resulting pRSII304 plasmid was sequenced

completely to verify the absence of spurious mutations. After the discovery of several disagreements with the Genbank sequence for pRS304 (NCBI gi: 416305), we also sequenced pRS304 with the same oligonucleotide primers for an accurate comparison. We subsequently determined the presence of errors in the Genbank sequence detailed in the Supporting Results.

The mutagenized *TRP1* marker was excised from pRSII304 by digesting with *Pml*I and *Dra*III, and ligated to pRS314, pRS404 and pRS414 cut with the same enzymes to generate pRSII314, pRSII404 and pRSII414, respectively. pRSII324 and pRSII424 were subsequently generated by ligating the 2.9 kb *Alw*NI-*Pfo*I fragment from pRSII423 containing the 2μ replication origin to pRSII304 and pRSII404, respectively, cut with *Alw*NI and *Pfo*I.

pRSII305/405 (*LEU2*) series: Starting with the *LEU2* marker of the plasmid YIplac128 (Gietz and Sugino 1988), we eliminated the *Cla*I site within the coding sequence by PCR-mediated site-directed mutagenesis (Table S1). A 1.1 kb fragment of the resulting *LEU2* allele spanning the mutagenized *Cla*I, *Kpn*I and *Eco*RI sites was excised from YIplac128 using a double digest at 60°C with *Bst*EII and *Bsr*GI. This fragment was ligated to pRS305, pRS315, pRS405 and pRS415 cut with the same two enzymes to generate pRSII305, pRSII315, pRSII405 and pRSII415, respectively. pRSII325 and pRSII425 were generated by ligating the 2.9 kb *Alw*NI-*Pfo*I fragment from pRSII423 containing the 2μ replication origin to pRSII305 and pRSII405, respectively, cut with *Alw*NI and *Pfo*I. It should be noted that removal of the *Kpn*I site within *LEU2* marker also caused the elimination of the overlapping *Age*I site.

pRSII306/406 (*URA3*) series: A 0.9 kb fragment of the *URA3* gene spanning the mutagenized *Pst*I site was excised from YIplac211 (Gietz and Sugino 1988) using *Nde*I and *Nsi*I. This fragment was ligated to circularized pGEM-T cut with the same two enzymes. The pGEM-T-*URA3* plasmid was then subject to site-directed mutagenesis to eliminate the *Apa*I site within the *URA3* coding sequence (Table S1). The mutagenized *URA3 Nde*I-*Nsi*I fragment was excised and subsequently ligated to pRS306, pRS316, pRS406 and pRS416 digested with *Nde*I and *Nsi*I to generate pRSII306, pRSII316, pRSII406 and pRSII416.

pRSII426 was constructed by first ligating the same *Nde*I-*Nsi*I fragment of $URA3^{\Delta PstI, ApaI}$ to pRS426 digested with *Nde*I and *Nsi*I, and then replacing the 0.65kb *Sna*BI-*Mfe*I section of the 2μ origin with the equivalent *Sna*BI-*Mfe*I fragment from YEplac195 (Gietz and Sugino 1988). To generate pRSII326, pRSII306 was first digested to completion with *Pfo*I and then partially digested with *Alw*NI; the remaining 2.9 kb fragment was subsequently ligated to the 2.9 kb *Alw*NI-*Pfo*I fragment from pRSII423 containing the 2μ replication origin.

pRSII309/409 (*HIS2*) series: The *HIS2* marker was originally amplified by PCR from YIpGAP2 (Sia *et al.* 1996) and TA cloned into pGEM-T-Easy as previously described (Chee and Haase 2010). The two *Bam*HI sites in *HIS2* were first eliminated sequentially by site-directed mutagenesis (Table S1). Next, the two *Xho*I sites were mutagenized in separate pGEM-T-Easy-$HIS2^{\Delta 2BamHI}$ clones (Table S1). pGEM-T-Easy-$HIS2^{\Delta 2BamHI, 2XhoI}$ was constructed by first excising the 1.1 kb *Xba*I-*Mfe*I fragment from

the clone containing the mutagenized *Xho*I site at nucleotide -9 in the *HIS2* gene. This fragment was then ligated to the second clone containing the mutagenized *Xho*I site at nucleotide +747 in *HIS2* after digestion with *Xba*I and *Mfe*I.

New pRS plasmids carrying the mutagenized *HIS2* were constructed as follows. The mutagenized *HIS2* gene, including 356 bp of 5' upstream sequence and 199 bp of 3' downstream sequence (-355 to +1208), was PCR-amplified using KOD HotStart polymerase with 5' *Nsi*I and 3' *Nde*I sites. The purified PCR product was digested overnight with *Nde*I and then for additional 3 hours with *Nsi*I. The digested PCR product was ligated to pRS306 cut with both *Nde*I and *Nsi*I to generate pRSII309; the 0.9 kb *Nde*I/*Nsi*I fragment excised from the *URA3* marker encompasses all of the protein-coding sequence. After sequencing, the *HIS2* marker was excised from pRSII309 by double digesting with *Nde*I and *Nsi*I, and subsequently ligated to pRS316, pRS406 and pRS416 cut with the same enzymes to generate pRSII319, pRSII409 and pRSII419. The 2μ episomal plasmids pRSII329 and pRSII429 were subsequently made by ligating the 2.9 kb *Alw*NI-*Pfo*I fragment from pRSII423 containing the 2μ replication origin to *Alw*NI and *Pfo*I digested pRSII309 and pRSII409, respectively.

Generalized pRS backbone vectors with a unique *Bgl*II site for inserting new yeast-selectable markers: pRS402 was restricted with *Bgl*II and *Nde*I to excise *ADE2* as well as the previously undocumented repeat sequence described in the main text's Results section. The remaining 3.3 kb backbone was treated with DNA polymerase I Klenow fragment (New England Biolabs, Beverly, MA, USA) to blunt the overhangs. The blunted ends were subsequently ligated to recircularize the backbone, resulting in a plasmid (pRS40BglII) with a single *Bgl*II site between the two pRS primer binding sites. The procedure of digestion, blunting and ligation was repeated with pRS412 and pRS422 to generate *CEN* and 2μ origin backbones (pRS41BglII and pRS42BglII, respectively) with the same *Bgl*II unique site. We went on to replace the 0.65 kb *Sna*BI-*Mfe*I fragment of the 2μ replication origin in pRS42BglII with with the *Sna*BI-*Mfe*I fragment from YEplac195 (Gietz and Sugino 1988) in order to eliminate the *Xba*I site within, hence generating pRSII42BglII).

To allow for the generation of pRS300 series vectors with customized markers, we replaced the the pBluescript II SK+ MCS in our pRS400 series backbone plasmids with the pBluescript KS+ MCS from pRS305. The replacement was carried out by excising the 1.2 kb *Ngo*MIV-*Alw*NI fragment encompassing the MCS from pRS305 and ligating it to pRS40BglII, pRS41BglII, pRS42BglII and pRSII42BglII double digested with the same two enzymes.

pRSII302/402 series (*ADE2*): The 2.3 kb *Bgl*II fragment containing the *ADE2* coding sequence along with 375 bp of upstream and 152 bp of downstream non-coding sequence was excised from pRS402 (Brachmann *et al.* 1998) and subcloned into pMCS5 (MoBiTech). The *ADE2* gene from pRS402 lacks the *Bgl*II site found at nucleotide position +593 in the *ADE2* sequence of *S. cerevisiae* strain S288C available from the *Saccharomyces* Genome Database (SGD, www.yeastgenome.org) (Brachmann *et al.* 1998). Mutagenesis of the two *Xba*I sites and the four *Hin*dIII sites in *ADE2* was carried out on two separate

pMCS5-*ADE2* clones: the first clone had the *Xba*I site at +7 and the *Hin*dIII site at +510 eliminated while the other sites were mutagenized in the second clone. pMCS5-$ADE2^{\Delta 4HindIII,\ 2XbaI}$ was subsequently constructed by first excising the 1.1 kb *Stu*I-*Xho*I fragment from the second clone; this fragment was ligated to the first clone after it had been digested with *Stu*I and *Xho*I.

The mutagenized *ADE2* gene was excised from pMCS5 by digesting with *Bgl*II and ligated to the Bgl*II*-digested backbone plasmids pRS40BglII, pRS41BglII and pRSII42BglII in order to construct pRSII402, pRSII412 and pRSII422, respectively. Ligation to pRS30BglII, pRS31BglII and pRSII32BglII was carried out to generate pRSII302, pRSII312 and pRSII322, respectively. Ligation products were screened to ensure a uniform orientation for the $ADE2^{\Delta 4HindIII,\ 2Xba}$ inserts.

pRSII308/408 series (*ADE1*): A 1.9 kb *Xho*I fragment originally isolated from pUC19-*ADE1* (NAGLEY *et al.* 1988) that contains the *ADE1* coding sequence along with 397 bp of upstream and 291 bp of downstream non-coding sequence was excised from pHV132, a plasmid designed for disrupting *STE4* with *ADE1* (a gift from M. Henar Valdivieso). This particular clone of *ADE1* does not contain the *Bam*HI site found at nucleotide position +162 in the *ADE1* genomic sequence from the SGD (NAGLEY *et al.* 1988). The excised *Xho*I fragment containing the *ADE1* gene was ligated to circularized pDrive (Qiagen) cut with *Xho*I.

PCR-based site-directed mutagenesis was used on pDrive-*ADE1* to eliminate the *Eco*RI and *Xba*I sites in the *ADE1* gene (Table S1). pDrive-$ADE1^{\Delta EcoRI,\ XbaI}$ was further mutagenized to eliminate the two *Sal*I sites found in the *ADE1* coding sequence in separate clones. pDrive-$ADE1^{\Delta BamHI,\ EcoRI,\ XbaI,\ 2SalI}$ was subsequently made by first excising the 1.1 kb *Bam*HI-*Hpa*I fragment from the clone containing the mutagenized *Bam*HI, *Eco*RI and *Sal*I sites lying upstream of the *Hpa*I site in ADE1. This fragment was then ligated to the second clone containing the mutagenized *Sal*I site at +854 in *ADE1* after double digestion with *Bam*HI and *Hpa*I.

The mutagenized *ADE1* gene was subsequently PCR-amplified using KOD HotStart polymerase with a minimal promoter based on the findings of Myasnikov et al. (MYASNIKOV *et al.* 1991) and 105 bp of 3' downstream sequence (-235 to +1026), flanked by *Bgl*II sites. The purified PCR product was digested with *Bgl*II, and then ligated to pRSII40BglII restricted with *Bgl*II to generate pRSII408. After sequencing to verify the absence of unwanted errors, the mutagenized *ADE1* was excised from pRSII408 and ligated to the same *Bgl*II-cut backbone plasmids used to generate the *ADE2* pRSII series. Ligation products were screened to ensure a uniform orientation for the $ADE1^{\Delta BamHI,\ EcoRI,\ XbaI,\ 2SalI}$ inserts.

Generalized pRS backbone vectors with a unique *Age*I site for inserting new yeast-selectable markers: pRS305, pRS315, pRS405, pRS415 and pRS425 were all digested sequentially with *Age*I at 37°C and then with *Tth*111I at 65°C to excise *LEU2*. The remaining backbones were treated with DNA polymerase I Klenow fragment (New England Biolabs, Beverly, MA, USA) to blunt the overhangs. The blunted ends were subsequently ligated to recircularize the backbone, resulting in a plasmid

with a single *Age*I site between the two pRS primer binding sites (pRS30AgeI, pRS31AgeI, pRS40AgeI, pRS41AgeI and pRS42AgeI, respectively). pRS32AgeI was constructed by excising the MCS of pRS42AgeI with *Alw*NI and *Ngo*MIV, and ligating the remainder of the plasmid to the 1.2 kb *Alw*NI-*Ngo*MIV fragment from pRS305. pRSII32AgeI and pRSII42AgeI were subsequently constructed by digesting pRS30AgeI and pRS40AgeI with *Alw*NI and *Pfo*I, followed by ligation to the 2.9 kb *Alw*NI-*Pfo*I fragment from pRSII423 containing the 2μ replication origin.

pRS40B: To construct pRS40B (pRS400-*ble*MX4), the *kan* gene was first excised from pRS400 by digesting sequentially with *Bgl*II and then with *Bsm*I. The *ble*MX4 cassette was excised from pUG66 by the same sequential *Bgl*II-*Bsm*I digest (GUELDENER *et al.* 2002) and ligated to pRS400 (*Bsm*I/*Bgl*II). It should be noted that although this *ble* gene has the same function as the *Streptoalloteichus hindustanus ble* gene (GATIGNOL *et al.* 1988) found in the plasmid pFA6a-*ble*MX6 constructed by Hentges and colleagues (HENTGES *et al.* 2005), it was originally isolated from the bacterial transposon Tn5 (GATIGNOL *et al.* 1987) and originates from *Klebsiella pneumoniae*; the two *ble* genes encode proteins that share only 4% amino acid sequence similarity.

pRS40H/N/P: To construct pRS40H (pRS400-*hph*MX4), pRS40N (pRS400-*nat*MX4) and pRS40P (pRS400-*pat*MX4), the *kan*MX4 module was first excised from pRS400 by digesting sequentially with *Asc*I and then with *Bsm*I. Using the same sequential *Asc*I-*Bsm*I digest, the *hph*MX4, *nat*MX4 and *pat*MX4 cassettes were excised from pAG32, pAG25 and pAG29 (GOLDSTEIN and MCCUSKER 1999), respectively, and ligated to pRS400 (*Asc*I/*Bsm*I).

***ADE2* deletion by PCR-based homologous recombination**

MX4 cassette PCR and yeast transformation: MX4 cassettes were first amplified by PCR as described in Materials & Methods using *ADE2*-specific primers (Table S3) and the plasmids pRS400, pRS40B, pRS40H, pRS40N and pRS40P (Table 2) as templates. Due to the high yield of PCR product obtained, each MX4 cassette was subsequently transformed into *S. cerevisiae* without prior cleanup or concentration. We used the same amount of PCR product that we analyzed by gel electrophoresis (Figure S2) to transform yeast. We advise against the use of excessive amounts of PCR product during transformation as doing so tends to increase the amount of background and lowers the efficiency of transformation (the number of drug-resistant transformants that actually carry the desired gene replacement) in our experience, similarly to transforming yeast with excessive amounts of plasmid DNA (GIETZ and SCHIESTL 2007). Following the heat shock step during yeast transformation (GIETZ and SCHIESTL 2007; GIETZ and WOODS 2001), yeast were resuspended in YEPD and allowed to recover at room temperature on a nutator for 3 hours before plating on the appropriate selective medium. Resistant colonies that appeared were subject to a second round of selection by repatching on fresh selective plates. While we have included our own recipes for selective media below, we advise users to determine kill curves for their particular yeast strain due to differences in susceptibility, and also to

lot-to-lot and manufacturer-specific variations in drug potency. Users seeking to delete additional genes with MX drug resistance cassettes in a strain already carrying an integrated MX cassette in its genome should consider employing multiple drug selection. Due to the identical *TEF* promoter and terminator sequences present in all MX cassettes, multiple drug selection will maintain selection for the previously used MX cassette and limit unwanted recombination with the new MX cassette to be transformed into the strain.

***kan*MX4 selection:** *kan*MX4 transformants were selected for by plating on YEPD supplemented with 200 μg/ml G418 sulfate (Calbiochem or Cellgro) a.k.a. Geneticin. Users who experience large amounts of background should note that the use of glycerol in place of dextrose as the carbon source during selection has been reported to reduce the number of untransformed G418-resistant colonies (HOLLENBERG 1982). Plate media containing G418 are stable for at least six months when stored at 4°C. G418 can also be used on minimal medium but yeast nitrogen base without ammonium sulfate must be used, and 1 g L-glutamate added per liter of medium (http://mgm.duke.edu/faculty/mccusker/lab/resources/) for selection to be efficient.

***ble*MX4 selection:** *ble*MX4 transformants were selected for by plating on YEPD (pH 7.0) supplemented with 50-150 μg/ml Zeocin (Invitrogen), a member of the bleomycin/phleomycin family of antibiotics. We have found that a yeast strain expressing Tn5 *ble,* SBY1143 (CHEE and HAASE 2010), can form colonies on YEPD (pH 7.0) containing as much as 300 μg/ml Zeocin. According to the manufacturer's protocol, incubating transformed yeast on ice for 1 hr following recovery prior to plating will reduce the number of untransformed Zeocin-resistant colonies; we have found this to be true in our experience but the total number of actual transformants is also reduced. According to the manufacturer, Zeocin selection can also be carried out on minimal medium. Although we have not tested Zeocin selection on minimal medium ourselves, a recent study on Zeocin selection using Pombe *minimal* glutamate medium in *Schizosaccharomyces pombe* may provide a useful guide (BENKO and ZHAO 2011). Zeocin is unstable and also light-sensitive so plate media containing Zeocin should be stored at 4°C in the dark for up to one month only; however, we have successfully used plates that were 5 weeks old.

Additionally, phleomycin selection of Tn5 *ble* transformants has been reported to be more efficient when glycerol is used as the carbon source instead of dextrose during selection (GATIGNOL *et al.* 1987). Optimal transformation efficiency with the Tn5 *ble* marker has also been reported after 6 h of recovery in non-selective medium (WENZEL *et al.* 1992). We have not, however, explicitly tested these suggested conditions with Zeocin selection.

***hph*MX4 selection:** *hph*MX4 transformants were selected for by plating on YEPD supplemented with 150-300 μg/ml hygromycin B (A.G. Scientific or Invivogen). Hygromycin B is light-sensitive, hence plate media containing hygromycin B should be stored at 4°C in the dark. We have found that the amount of background usually increases significantly if the plates are over

a month old. Like G418, hygromycin B can be used on minimal medium but without ammonium sulfate and with 1 g L-glutamate added per liter of medium (http://mgm.duke.edu/faculty/mccusker/lab/resources/).

***nat*MX4 selection:** *nat*MX4 transformants were selected for by plating on YEPD supplemented with 85-100 μg/ml nourseothricin sulfate (Jena Bioscience), also known as clonNAT. In our experience, plate media containing nourseothricin when stored at 4°C are more stable than plate media containing either Zeocin or hygromycin B. We have successfully used nourseothricin plates that were three months old. Interestingly, nourseothricin selection has been reported to work very well on minimal media with *Schizosaccharomyces pombe*, making it suitable for double selection in tandem with a prototrophic marker (HENTGES *et al.* 2005). For *S. cerevisiae*, minimal media containing nourseothricin should be made without ammonium sulfate and with L-glutamate added, as indicated for G418 and hygromycin B (http://mgm.duke.edu/faculty/mccusker/lab/resources/).

***pat*MX4 selection**: *pat*MX4 transformants were first spun down and resuspended in sterile water before being plated on synthetic proline dextrose plates (0.67% yeast nitrogen base with 0.1% L-proline acid as nitrogen source, 2% dextrose) with 200 μg/ml bialaphos (Gold Biotechnology) added. Plate media containing bialaphos are unstable and should be stored at 4°C for up to one month only (GOLDSTEIN and MCCUSKER 1999). Users should note that bialaphos selection works best with prototrophic yeast due to the stringent media requirements and that reliable selection conditions for auxotrophic yeast have yet to be determined.

Verifying *ADE2* deletion: Ade$^-$ (adenine auxotrophic) transformants were subsequently identified by replica plating drug-resistance isolates on synthetic complete medium with 2% dextrose but lacking adenine. Genomic DNA was isolated from both wild-type S288C and Ade$^-$ transformants. Diagnostic PCR was carried out to verify the presence of the MX4 cassette at the *ADE2* genomic locus using *ADE2*-flanking primers (*ADE2* upstream FP: 5'- GGTGCG TAAAATCGTTGGATCTCT-3' and *ADE2* downstream RP: 5'-GGACACCTGTAAGCGTTGATTTCT-3'). Successful replacement of *ADE2* was scored for by changes in PCR product size as detected by agarose gel electrophoresis; however, as *ADE2* replacement with the *hph*MX4 cassette from pRS40H does not cause a detectable change in size of the diagnostic PCR product, an *Afl*II digest was performed which only cuts *ADE2* and not *hph*MX4.

Results

***ADE2* deletion by PCR-based homologous recombination using pRS40B/H/N/P:** The new pRS400-derived plasmids were tested by using them to replace the *ADE2* gene in the yeast genome with the respective PCR-amplified MX4 drug resistance cassettes (Figure S2). pRS400 (BRACHMANN *et al.* 1998) was used as a positive control. pRS40B, pRS40H and pRS40N were used successfully to replace *ADE2* in the wild-type strain S288C (MORTIMER and JOHNSTON 1986) (Table S5). All resulting Ade^{-} transformants were verified by PCR to carry the respective MX4 cassette at the *ADE2* genomic locus (data not shown). Unfortunately, the results for *pat*MX4 transformations were inconsistent due to unforeseen difficulties with our bialaphos media. We subsequently learnt that other users have also experienced difficulties with bialaphos selection during *pat*MX4 transformations (P. Gold, 2012, personal communication) using the original pAG29 plasmid (GOLDSTEIN and MCCUSKER 1999).

Significant errors in publicly available nucleotide sequences for yeast plasmids

pRS303/403 (*HIS3*) series: As has been pointed out on VectorDB (http://genome-www.stanford.edu/vectordb/vector.html), the *HIS3* marker found in the pRS3x3/4x3 plasmids matches the *HIS3* genomic sequence found on the Saccharomyces Genome Database (SGD); however, the current Genbank sequences for these plasmids indicates that the *HIS3* marker sequence is instead identical to that of the annotated *HIS3* gene sequence found on Genbank (X03245). Hence, there is a previously undocumented *Sfi*I site present in the *HIS3* marker of the pRS3x3/4x3 plasmids. Additionally, the *Nde*I site within the *HIS3* marker sequence is unique as we have determined that the second *NdeI* site flanking the 3' end of *HIS3* in the Genbank sequences of pRS303 and pRS313 (Table S4) does not exist.

pRS304/404 (*TRP1*) series: The sequences immediately flanking the *TRP1* marker in the pRS3x4 and pRS4x4 plasmids are missing insertions of six and four nucleotides, respectively. As a consequence, the *Alf*I site that flanks the 3' end of the *TRP1* marker (closest to the MCS) in the pRS304 and pRS314 Genbank sequences (Table S6) does not exist.

pRS305/405 (*LEU2*) series: The *LEU2* marker found in the pRS series plasmids matches the *LEU2* genomic sequence found on SGD. Additionally, there is an undocumented *Age*I site that flanks the *LEU2* marker on its 5' end (closest to the MCS) in the pRS3x5/4x5 plasmids; the *Xho*I site that flanks this end of the *LEU2* marker in the pRS305 and pRS315 Genbank sequences (Table S6) does not exist.

pRS306/406 (*URA3*) series: The *Bse*YI and *Gsa*I sites closest to the *Kpn*I site of the MCS in the pRS306 and pRS316 Genbank sequences (Table S6) do not exist.

pRS400 (*kan*MX4): As shown in Figure 4, the *kan*MX4 cassette in this plasmid is oriented in the opposite direction to that which its Genbank seqeuence (Table S6) indicates.

pAG25: Nucleotide #89 in this plasmid's EUROSCARF sequence (Table S6) is C and not T.

pAG29: We determined ten points of disagreement with the nucleotide sequence of the *pat*MX4 cassette in the EUROSCARF for pAG29 (Table S6), including two additional nucleotides that form part of an additional *Nco*I site at nucleotide #458 (Figure 4C). The *Nco*I site missing from the plasmid sequence was introduced during construction of the *pat*MX4 cassette by the addition of a glycine codon between the ATG start codon and the second codon of the *pat* gene, which encodes a serine residue (GOLDSTEIN and MCCUSKER 1999). Its omission from the plasmid sequence causes a frameshift mutation in the *pat* coding sequence, leading to a truncated ORF being identified by sequence analysis software that encodes a 151 amino acid polypeptide whereas the actual ORF encodes a 184 amino acid protein (*Streptomyces viridochromogenes* phosphinothricin N-acetyltransferase with an additional glycine residue at its N-terminus following the initiating methionine).

pAG32: Nucleotide #89 in the EUROSCARF sequence (Table S6) is C and not T and nucleotide #284 is G and not A. Thus, the *Pst*I site that encompasses #284 does not exist.

pUG66: There are three single nucleotide differences with the EUROSCARF sequence (Table S6) and a six nucleotide insertion at nucleotide #489. As a result, a previously undocumented *Bsa*BI site (albeit one that overlaps with a *dam* methylation site) exists at #533.

LITERATURE CITED

BENKO, Z., and R. Y. ZHAO, 2011 Zeocin for selection of *ble*MX6 resistance in fission yeast. Biotechniques **51:** 57-60.

BRACHMANN, C. B., A. DAVIES, G. J. COST, E. CAPUTO, J. LI *et al.*, 1998 Designer deletion strains derived from *Saccharomyces cerevisiae* S288C: a useful set of strains and plasmids for PCR-mediated gene disruption and other applications. Yeast **14:** 115-132.

CHEE, M. K., and S. B. HAASE, 2010 B-Cyclin/CDKs regulate mitotic spindle assembly by phosphorylating kinesins-5 in budding yeast. PLoS Genetics **6:** e1000935. doi:1000910.1001371/journal.pgen.1000935.

GATIGNOL, A., M. BARON and G. TIRABY, 1987 Phleomycin resistance encoded by the *ble* gene from transposon Tn 5 as a dominant selectable marker in *Saccharomyces cerevisiae*. Molecular and General Genetics **207:** 342-348.

GATIGNOL, A., H. DURAND and G. TIRABY, 1988 Bleomycin resistance conferred by a drug-binding protein. FEBS Letters **230:** 171-175.

GIETZ, R. D., and R. H. SCHIESTL, 2007 High-efficiency yeast transformation using the LiAc/SS carrier DNA/PEG method. Nature Protocols **2:** 31-34.

GIETZ, R. D., and A. SUGINO, 1988 New yeast-*Escherichia coli* shuttle vectors constructed with in vitro mutagenized yeast genes lacking six-base pair restriction sites. Gene **74:** 527-534.

GIETZ, R. D., and R. A. WOODS, 2001 Genetic transformation of yeast. Biotechniques **30:** 816-820.

GOLDSTEIN, A. L., and J. H. MCCUSKER, 1999 Three new dominant drug resistance cassettes for gene disruption in *Saccharomyces cerevisiae*. Yeast **15:** 1541-1553.

GUELDENER, U., J. HEINISCH, G. KOEHLER, D. VOSS and J. HEGEMANN, 2002 A second set of loxP marker cassettes for Cre-mediated multiple gene knockouts in budding yeast. Nucleic Acids Research **30:** e23.

HARTLEY, J. L., and J. E. DONELSON, 1980 Nucleotide sequence of the yeast plasmid. Nature **286:** 860-865.

HENTGES, P., B. DRIESSCHE, L. TAFFOREAU, J. VANDENHAUTE and A. M. CARR, 2005 Three novel antibiotic marker cassettes for gene disruption and marker switching in *Schizosaccharomyces pombe*. Yeast **22:** 1013-1019.

HOLLENBERG, C., 1982 Cloning with 2-micrometer DNA vectors and the expression of foreign genes in Saccharomyces cerevisiae. Current Topics in Microbiology and Immunology **96:** 119-144.

MAHADEVAN, S., T. R. RAGHUNAND, S. PANICKER and K. STRUHL, 1997 Characterisation of 3' end formation of the yeast *HIS3* mRNA. Gene **190:** 69-76.

MORTIMER, R. K., and J. R. JOHNSTON, 1986 Genealogy of principal strains of the yeast genetic stock center. Genetics **113:** 35-43.

MYASNIKOV, A. N., K. V. SASNAUSKAS, A. A. JANULAITIS and M. N. SMIRNOV, 1991 The *Saccharomyces cerevisiae ADE1* gene: structure, overexpression and possible regulation by general amino acid control. Gene **109:** 143-147.

NAGLEY, P., L. B. FARRELL, D. P. GEARING, D. NERO, S. MELTZER *et al.*, 1988 Assembly of functional proton-translocating ATPase complex in yeast mitochondria with cytoplasmically synthesized subunit 8, a polypeptide normally encoded within the organelle. Proceedings of the National Academy of Sciences, USA **85:** 2091-2095.

SIA, R. A., H. A. HERALD and D. J. LEW, 1996 Cdc28 tyrosine phosphorylation and the morphogenesis checkpoint in budding yeast. Molecular Biology of the Cell **7:** 1657-1666.

SIKORSKI, R. S., and P. HIETER, 1989 A system of shuttle vectors and yeast host strains designed for efficient manipulation of DNA in *Saccharomyces cerevisiae*. Genetics **122:** 19-27.

WENZEL, T. J., A. MIGLIAZZA, H. Y. STEENSMA and J. A. VAN DEN BERG, 1992 Efficient selection of phleomycin-resistant *Saccharomyces cerevisiae* transformants. Yeast **8:** 667-668.
