# Supplementary material for: New and Redesigned pRS Plasmid Shuttle Vectors for Genetic Manipulation of Saccharomyces cerevisiae
Source: G3 (Bethesda). 2012 May 1;2(5):515–26. doi: 10.1534/g3.111.001917 (PMC3362935; doi:10.1534/g3.111.001917)
Supplement: Supporting Information [file supp_2.5.515_FigureS1.pdf]

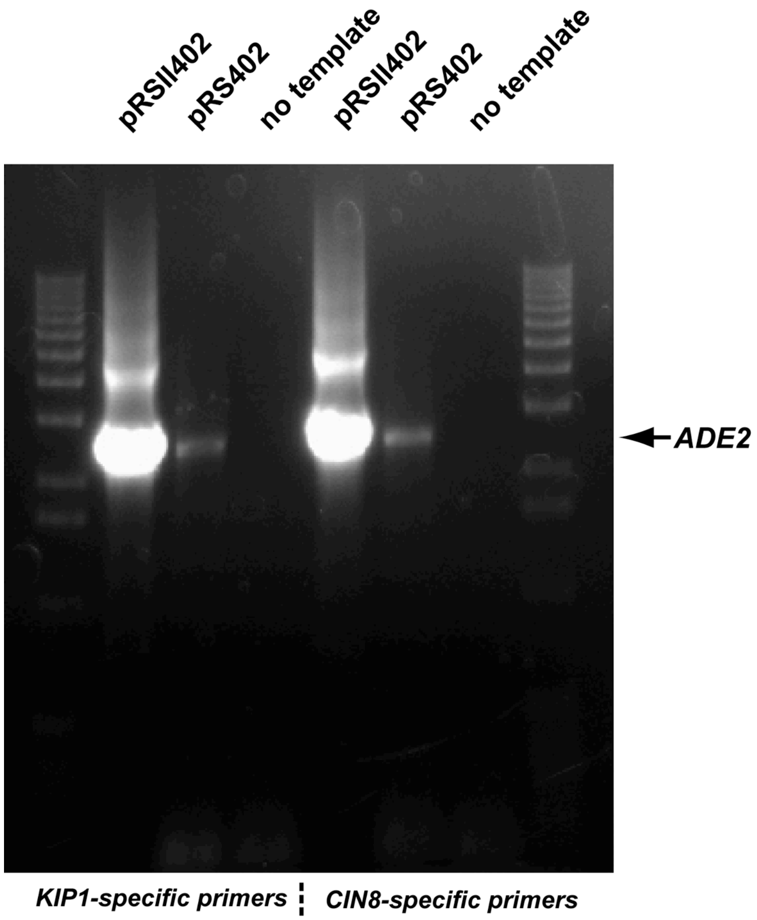

Figure S1 PCR amplification of *ADE2* marker for targeted gene replacement with either pRSII402 or pRS402 as the template. pRS402 contains two pRS reverse primer binding sites, including one previously undocumented site that lies between the pRS forward primer binding site and the *ADE2* marker. The extra undocumented site was removed from pRSII402. Sequences of the *KIP1*- and *CIN8*-specific oligonucleotide primers used are listed in Table S3. Control PCRs lacking a template plasmid were run to demonstrate specificity. 5.0 μl of each reaction was used for agarose gel electrophoresis; 0.5 μg of Invitrogen 1 kb DNA ladder was run on the same gel.
