# Supplementary material for: New and Redesigned pRS Plasmid Shuttle Vectors for Genetic Manipulation of Saccharomyces cerevisiae
Source: G3 (Bethesda). 2012 May 1;2(5):515–26. doi: 10.1534/g3.111.001917 (PMC3362935; doi:10.1534/g3.111.001917)
Supplement: Supporting Information [file supp_2.5.515_FigureS2.pdf]

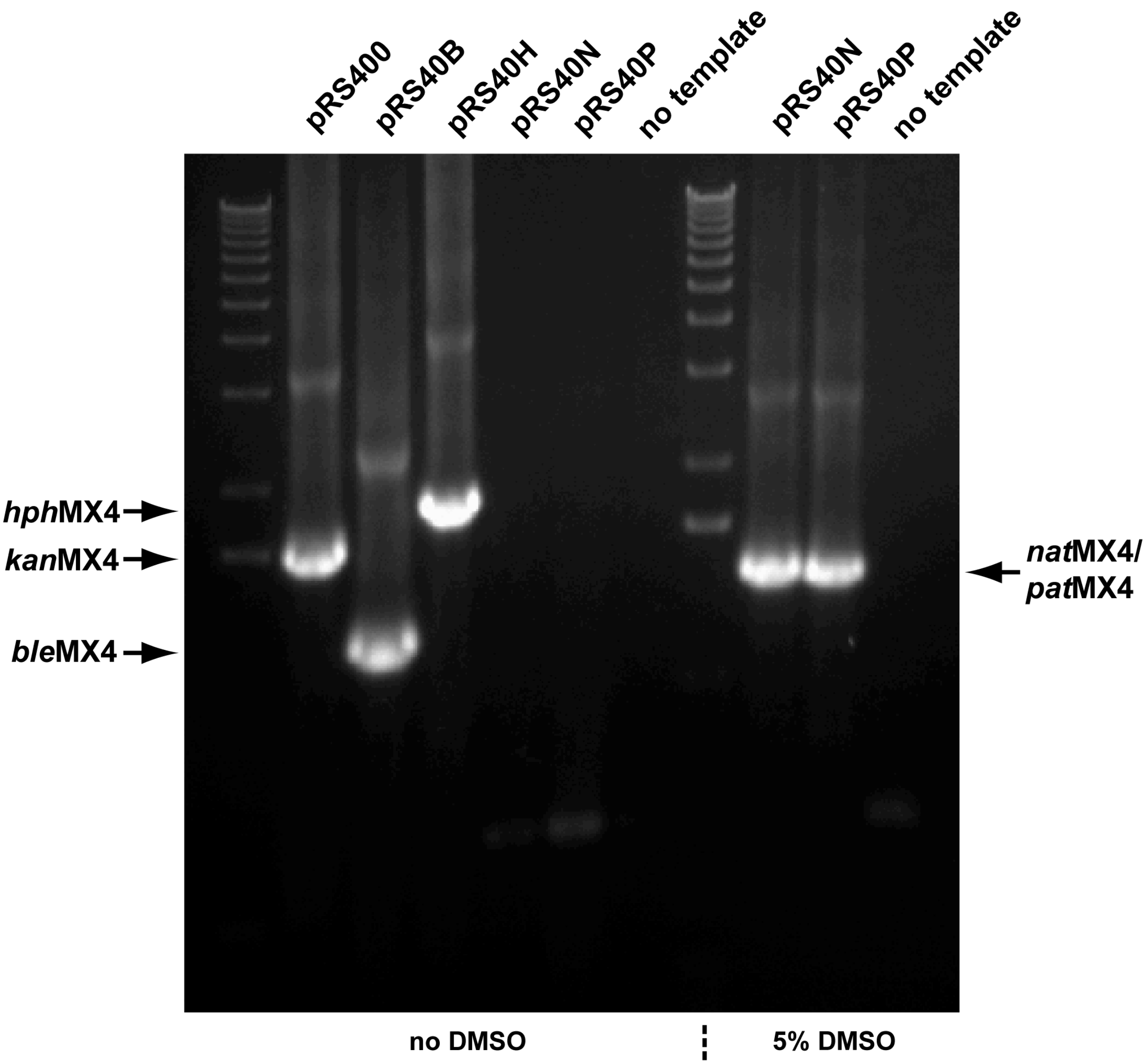

Figure S2 PCR amplification of MX4 markers from pRS400 and its new derivatives for targeted replacement of *ADE2* in the *S. cerevisiae* genome. Aside from pRS400 (*kan*MX4), the other plasmid templates are listed in Table 2 with the corresponding drug resistance genes. The sequences of the *ADE2*-specific oligonucleotide primers used are listed in Table S3. As noted in Materials & Methods, the addition of 5% DMSO to the PCR is required to amplify *nat*MX4 and *pat*MX4. Control PCRs lacking a template plasmid were run to demonstrate specificity. For the PCRs containing 5% DMSO, 2.0 μl was used for agarose gel electrophoresis while 5.0 μl was used from all the other PCRs; 0.5 μg of Invitrogen 1 kb DNA ladder was run on the same gel.
