# Supplementary material for: New and Redesigned pRS Plasmid Shuttle Vectors for Genetic Manipulation of Saccharomyces cerevisiae
Source: G3 (Bethesda). 2012 May 1;2(5):515–26. doi: 10.1534/g3.111.001917 (PMC3362935; doi:10.1534/g3.111.001917)
Supplement: Supporting Information [file supp_2.5.515_TableS1.pdf]

Table S1 Common restriction sites found in both yeast prototrophic marker sequences and the pBluescript/pBluescript II MCS[a]

Marker gene	Restriction enzyme recognition site											
	*Kpn*I	*Apa*I	*Xho*I	*Sal*I	*Cla*I	*Hin*dIII	*Eco*RV	*Eco*RI	*Pst*I	*Bam*HI	*Xba*I	*Bst*XI
ADE2	-	-	-	-	-	2	1	-	-	-	4	1
HIS3	1	-	-	-	-	2	-	-	1	-	-	1
TRP1	-	-	-	-	-	1	1	-	-	-	1	1
LEU2	1	-	-	-	1	-	1	1	-	-	-	1
URA3	-	1[b]	-	-	-	-	1	-	1	-	-	-
ADE1	-	-	-	2	-	-	-	1	-	1	1	-
HIS2	-	-	2	-	-	-	-	-	-	2	-	-

The number of times that each site occurs within the respective marker gene sequence is indicated in each column.

[a] The pBluescript KS+ MCS found in pRS/pRSII300 series plasmids is:
5'*Sac*I-*Bst*XI-*Sac*II-*Eag*I-*Not*I-*Xba*I-*Spe*I-*Bam*HI-*Sma*I-*Pst*I-*Eco*RI-*Eco*RV-*Hin*dIII-*Cla*I-*Sal*I-*Xho*I-*Apa*I-*Kpn*I-3'
The pBluescript II SK+ MCS found in pRS/pRSII400 series plasmids is:
5' *Kpn*I-*Apa*I-*Xho*I-*Sal*I-*Cla*I-*Hin*dIII-*Eco*RV-*Eco*RI-*Pst*I-*Sma*I-*Bam*HI-*Spe*I-*Xba*I-*Eag*I-*Not*I-*Bst*XI-*Sac*II-*Sac*I-3'

[b] Although the *Apa*I site in *URA3* overlaps with a *dcm* methylation site, plasmid DNA isolated from DH5α *dcm*$^{+}$ bacteria is still cleaved at this site by *Apa*I.
