# Supplementary material for: New and Redesigned pRS Plasmid Shuttle Vectors for Genetic Manipulation of Saccharomyces cerevisiae
Source: G3 (Bethesda). 2012 May 1;2(5):515–26. doi: 10.1534/g3.111.001917 (PMC3362935; doi:10.1534/g3.111.001917)
Supplement: Supporting Information [file supp_2.5.515_TableS2.pdf]

Table S2 Restriction sites targeted for removal in yeast auxotrophic marker sequences and oligonucleotide primers used for site-directed mutagenesis

Marker	Restriction site	Coordinates relative to translation start	Mutagenic oligonucleotide primers
ADE2	*Xba*I	+7	FP: 5'-CAA TCAAGTATGGATTCAAGAACAGTTGGTATA TTAGG-3'<br>RP: 5'-CCTAATATACCAACTGTTCTTGAATCCATACTTGATTG-3'
ADE2	*Hin*dIII	+510	FP: 5'-GGAAATGATTCCGGAAGCA TTGGAAGTACT GAAGG-3'<br>RP: 5'-CCTTCAGTACTTCCAATGCTTCCGGAATCATTTCC-3'
ADE2	*Xba*I	+1057	FP: 5'-GTACTTA TATGGAAAAGAGTCAAGACCTAACAGAAAA GTAGG-3'<br>RP: 5'-CCTACTTTTCTGTTAGGTCTTGACTCTTTTCCATATAAGTAG-3'
ADE2	*Xba*I	+1476	FP: 5'-CGTA AAAGGTTCTTGTCTTGATGGA GTAGATTCT TTAC-3'<br>RP: 5'-GTAAAGAATCTACTCCATCAAGACAAGAACCTTTTACG-3'
ADE2	*Hin*dIII<br>*Xba*I	+1695<br>+1701	FP: 5'-GAAACTGTCGGTTACGAAGCA TATCTT GAAAACAAGT AAT ATATAAG-3'<br>RP: 5'-CTTATATATTACTTGTTTTCAAGATATGCTTCGTAACCGACAGTTTC-3'
HIS3	*Hin*dIII	+306	5'-FP: GATTGCTCTCGGTCAAGCATTTAAAGAGGCCCTA-3'<br>RP: 5'-TAGGGCCTCTTTAAATGCTTGACCGAGAGCAATC-3'
HIS3	*Hin*dIII	+496	FP: 5'-CGCATTTTCTTGAAAGTTTTGCAGAGGCTAGCAG-3'<br>RP: 5'-CTGCTAGCCTCTGCAAAACTTTCAAGAAAATGCG-3'
HIS3	*Kpn*I	+627	FP: 5'-CTCGCCCAATGGTACAAACGATGTTCCCTCCA-3'<br>RP: 5'-TGGAGGGAACATCGTTTGTACCATTGGGCGAG-3'
HIS3	*Pst*I	+688 (3'UTR)	FP: 5'-GTGACACCGATTATTTAAAGTTGCAGCATACGATATATATACATGTG-3'<br>RP: 5'-CACATGTATATATATCGTATGCTGCAACTTTAAATAATCGGTGTCAC-3'
TRP1	*Xba*I	+85	Gietz and Sugino, 1988
TRP1	*Hin*dIII	+514	FP: 5'-GGCAAGAGA GCCCCGAGAG CTTACATTTT ATGTTAGC-3'<br>RP: 5'-GCTAACATAAAATGTAAGCTCTCGGGGCTCTCTTGCC-3'
LEU2	*Kpn*I	+252	Gietz and Sugino, 1988
LEU2	*Eco*RI	+638	Gietz and Sugino, 1988
URA3	*Pst*I	-17 (5'UTR)	Gietz and Sugino, 1988
ADE1	*Bam*HI	+162	Nagley *et al*., 1988
ADE1	*Sal*I	+230	FP: 5'-CGATGTTCGTA ATCATTTG GTAGACATCGCCCCAGGTAAGAC-3'<br>RP: 5'-GTCTTACCTGGGGCGATGTCTACCAAATGATTACGAACATCG-3'
ADE1	*Eco*RI	+656	FP: 5'-CG CAGACACTAAATTCGAATTTGGTATTGACG AAAAGACC-3'<br>RP: 5'-GGTCTTTTCGTCAATACCAAATTCGAATTTAGTGTCTGCG-3'
ADE1	*Xba*I	+722	FP: 5'-GCTAACGCCAGACTCCTCAAGATTCTGGAACGGTG-3'<br>RP: 5'-CACCGTTCCAGAATCTTGAGGAGTCTGGCGTTAGC-3'
ADE1	*Sal*I	+854	FP: 5'-CCCCAAGACATTGTAGACAGGACAAGGGCC-3'<br>RP: 5'-GGCCCTTGTCCTGTCTACAATGTCTTGGGG-3'
HIS2	*Xho*I	-8 (5'UTR)	FP: 5'-TCAGTAAAAATCCTCAAGGTCATGCACTCACAC-3'

			RP: 5’-GTGTGAGTGCATGACCTTGAGGATTTTTACTGA-3’
HIS2	*Bam*HI	+402	FP: 5’-TCACGTCAACGGGATACCTATTGATTTCGAC-3’
			RP: 5’-GTCGAAATCAATAGGTATCCCGTTGACGTGA-3’
HIS2	*Xho*I	+749	FP: 5’-GCATTAAGAAAGCGCCTGGAGGAGCCGTACCCC-3’
			RP: 5’-GGGGTACGGCTCCTCCAGGCGCTTTCTTAATGC-3’
HIS2	*Bam*HI	+803	FP: 5’-CAAGAAGCACTGTGGCTCCAGATTTGTTCTA-3’
			RP: 5’-TAGAACAAATCTGGAGCCACAGTGCTTCTTG-3’

FP = forward primer; RP = reverse primer
Mutagenized nucleotides are underlined
