# Supplementary material for: New and Redesigned pRS Plasmid Shuttle Vectors for Genetic Manipulation of Saccharomyces cerevisiae
Source: G3 (Bethesda). 2012 May 1;2(5):515–26. doi: 10.1534/g3.111.001917 (PMC3362935; doi:10.1534/g3.111.001917)
Supplement: Supporting Information [file supp_2.5.515_TableS3.pdf]

Table S3 Oligonucleotide primers used to test amplification of yeast-selectable marker sequences from pRS/pRSII plasmids

Primer name	Primer sequence
*kip1*Δ::pRS marker FP	5'-GCGCTTCCCTCACTAAATATGGCGAGATAGTTAAACAATCCAGATTGTACTGAGAGTGC-3'
*kip1*Δ::pRS marker RP	5'-TATAGTGATACAAATATTTTACAATGGCTATATCCCCTTACCTTACGCATCTGTGCGG-3'
*cin8*Δ::pRS marker FP	5'-TATAAAAGCGCAAAAAATACAACAAGAAAGAATTTGTTTGCAGATTGTACTGAGAGTGC-3'
*cin8*Δ::pRS marker RP	5'-TAGTTTGAATATATATTCGACTGAAAGGCAATATCAACTACCTTACGCATCTGTGCGG-3'
*ade2*Δ::pRS marker FP	5'-CAATCAAGAA AAACAAGAAAATCGGACAAAACAATCAAGTCAGATTGTACTGAGAGTGC-3'
*ade2*Δ::pRS marker RP	5'-ATTATTTGCTGTACAAGTATATCAATAAACTTATATATTACCTTACGCATCTGTGCGG-3'

Sequences identical to the pRS backbone are underlined
