# Supplementary material for: New and Redesigned pRS Plasmid Shuttle Vectors for Genetic Manipulation of Saccharomyces cerevisiae
Source: G3 (Bethesda). 2012 May 1;2(5):515–26. doi: 10.1534/g3.111.001917 (PMC3362935; doi:10.1534/g3.111.001917)
Supplement: Supporting Information [file supp_2.5.515_TableS4.pdf]

Table S4 Oligonucleotide primers used to sequence yeast plasmids

Sequencing primer name	Primer sequence
pRS forward	5’-CAGATTGTACTGAGAGTGC-3’
pRS reverse	5’-CCTTACGCATCTGTGCGG-3’
M13 reverse (-20)	5’-CAGGAAACAGCTATGACC-3’
M13 forward	5’-TGTAAAACGACGGCCAGT-3’
SP6	5’-CCGGGAGCTGCATGTGTCAGAGG-3’
T7	5’- TAATACGACTCACTATAGGG-3’
pGEX 3’	5’-TACGATTTAGGTGACACTATAG-3’
bla 5’ forward primer	5’-GGAAGAGTATGAGTATTCAACA-3’
bla 3’ reverse primer	5’-GTCTGACAGTTACCAATGCT-3’
bla 5’ reverse primer	5’-GGCGACACGGAA ATGTTGAA-3’
bla 3’ forward primer	5’-CCTCACTGATTAAGCATTGGTA-3’
ADE2 internal seq 1	5’-GCAGCAAACAGGCTCAACATT-3’
ADE2 internal seq 2	5’-CCTGCTAGAGTTCCGGACT-3’
TRP1 internal seq forward	5’-GGCAGCTTGGAGTATGTCT-3’
TRP1 internal seq reverse	5’-CCTGTCCCACCTGCTTCT-3’
LEU2 internal seq 1	5’-CCAATAGGTGGTTAGCAATG-3’
LEU2 internal seq 2	5’-CGCTTGGGATAGTGAACAATA-3’
LEU2 internal seq reverse	5’-GGCAGAATCAATCAATTGATG-3’
