# Supplementary material for: New and Redesigned pRS Plasmid Shuttle Vectors for Genetic Manipulation of Saccharomyces cerevisiae
Source: G3 (Bethesda). 2012 May 1;2(5):515–26. doi: 10.1534/g3.111.001917 (PMC3362935; doi:10.1534/g3.111.001917)
Supplement: Supporting Information [file supp_2.5.515_TableS5.pdf]

Table S5 Total numbers of S288C *ade2Δ* transformants obtained with MX4 casettes amplified from pRS400-derived plasmids

PCR product used for transformation	Total number of drug-resistant transformants isolated[a]	Number of drug-resistant, *ade2*Δ transformants
*ade2*Δ::*ble*MX4	57	11
	24[b]	1[b]
*ade2*Δ::*hph*MX4	8	3
*ade2*Δ::*nat*MX4	4	2
ade2Δ::*kan*MX4	14	6

Numbers shown are the totals obtained from two independent transformations.
[a] Following two rounds of drug selection to reduce false positives
[b] Numbers obtained when cells were incubated on ice for 1 h after recovery in YEPD prior to plating
