# Supplementary material for: New and Redesigned pRS Plasmid Shuttle Vectors for Genetic Manipulation of Saccharomyces cerevisiae
Source: G3 (Bethesda). 2012 May 1;2(5):515–26. doi: 10.1534/g3.111.001917 (PMC3362935; doi:10.1534/g3.111.001917)
Supplement: Supporting Information [file supp_2.5.515_TableS6.pdf]

Table S6 Existing publicly available sequences for yeast shuttle vectors in need of correction

Plasmid name	Database where sequence is stored	Current accession/version number	Last updated
pRS303	Genbank	U03435.1	Sep 14, 1995
pRS313	Genbank	U03439.1	Sep 14, 1995
pRS304	Genbank	U03436.1	Sep 14, 1995
pRS314	Genbank	U03440.1	Sep 14, 1995
pRS305	Genbank	U03437.1	Sep 14, 1995
pRS315	Genbank	U03441.1	Sep 14, 1995
pRS306	Genbank	U03438.1	Sep 14, 1995
pRS316	Genbank	U03442.1	Sep 14, 1995
pRS402	Genbank	U93717.1	Sep 3, 1997
pRS412	Genbank	U93718.1	Sep 3, 1997
pRS422	Genbank	U93719.1	Sep 3, 1997
pRS403	Genbank	U03443.1	May 24, 1995
pRS413	Genbank	U03447.1	May 24, 1995
pRS423	Genbank	U03454.1	May 24, 1995
pRS404	Genbank	U03444.1	May 24, 1995
pRS414	Genbank	U03448.1	May 24, 1995
pRS424	Genbank	U03453.1	May 24, 1995
pRS405	Genbank	U03445.1	May 24, 1995
pRS415	Genbank	U03449.1	May 24, 1995
pRS425	Genbank	U03452.1	May 24, 1995
pRS406	Genbank	U03451.1	May 24, 1995
pRS416	Genbank	U03450.1	May 24, 1995
pRS426	Genbank	U03451.1	May 24, 1995
pRS400	Genbank	U93713.1	Sep 3, 1997
pAG25	EUROSCARF[a]	P30104	N.A.

pAG29	EUROSCARF	P30105	N.A.
pAG32	EUROSCARF	P30106	N.A.
pUG66	Genbank/EUROSCARF	AF298794.1/ P30116	Mar 11, 2002

[a] European *Saccharomyces cerevisiae* Archive for Functional Analysis
